# Identification and experimental validation of key m6A modification regulators as potential biomarkers of osteoporosis

**DOI:** 10.3389/fgene.2022.1072948

**Published:** 2023-01-06

**Authors:** Yanchun Qiao, Jie Li, Dandan Liu, Chenying Zhang, Yang Liu, Shuguo Zheng

**Affiliations:** Department of Preventive Dentistry, Peking University School and Hospital of Stomatology & National Center of Stomatology & National Clinical Research Center for Oral Diseases & National Engineering Laboratory for Digital and Material Technology of Stomatology & Beijing Key Laboratory of Digital Stomatology & Research Center of Engineering and Technology for Computerized Dentistry Ministry of Health & NMPA Key Laboratory for Dental Materials, Beijing, China

**Keywords:** osteoporosis, bone metabolism, M6A, RNA modification, biomarker, osteoclast

## Abstract

Osteoporosis (OP) is a severe systemic bone metabolic disease that occurs worldwide. During the coronavirus pandemic, prioritization of urgent services and delay of elective care attenuated routine screening and monitoring of OP patients. There is an urgent need for novel and effective screening diagnostic biomarkers that require minimal technical and time investments. Several studies have indicated that N6-methyladenosine (m6A) regulators play essential roles in metabolic diseases, including OP. The aim of this study was to identify key m6A regulators as biomarkers of OP through gene expression data analysis and experimental verification. GSE56815 dataset was served as the training dataset for 40 women with high bone mineral density (BMD) and 40 women with low BMD. The expression levels of 14 major m6A regulators were analyzed to screen for differentially expressed m6A regulators in the two groups. The impact of m6A modification on bone metabolism microenvironment characteristics was explored, including osteoblast-related and osteoclast-related gene sets. Most m6A regulators and bone metabolism-related gene sets were dysregulated in the low-BMD samples, and their relationship was also tightly linked. In addition, consensus cluster analysis was performed, and two distinct m6A modification patterns were identified in the low-BMD samples. Subsequently, by univariate and multivariate logistic regression analyses, we identified four key m6A regulators, namely, *METTL16*, *CBLL1*, *FTO*, and *YTHDF2*. We built a diagnostic model based on the four m6A regulators. *CBLL1* and *YTHDF2* were protective factors, whereas *METTL16* and *FTO* were risk factors, and the ROC curve and test dataset validated that this model had moderate accuracy in distinguishing high- and low-BMD samples. Furthermore, a regulatory network was constructed of the four hub m6A regulators and 26 m6A target bone metabolism-related genes, which enhanced our understanding of the regulatory mechanisms of m6A modification in OP. Finally, the expression of the four key m6A regulators was validated *in vivo* and *in vitro*, which is consistent with the bioinformatic analysis results. Our findings identified four key m6A regulators that are essential for bone metabolism and have specific diagnostic value in OP. These modules could be used as biomarkers of OP in the future.

## 1 Introduction

Osteoporosis (OP) is a systemic skeletal disease characterized by increased fracture risk and decreased bone density or bone strength that occurs widely in postmenopausal women (Miller, 2016). The prevalence of OP increases with age, from 19.57% in women aged 50–59 years to 56.10% in women aged 80 years and older, and it will continue to rise with the aging of the population in China (Chen et al., 2016). Traditionally, bone mineral density (BMD) measured by dual X-ray absorptiometry (DXA) is used to diagnose OP, assess fracture risk, and monitor changes in BMD over time (Chun, 2011). However, DXA presents some disadvantages, namely, that accessibility to DXA is limited in many locations (Curtis et al., 2017). The rapid spread of the COVID-19 pandemic makes it more difficult to monitor BMD frequently during OP therapy, as medical resources are diverted from chronic disease care to combat the pandemic. In addition, errors in DXA scans/reports are common due to difficulties in the maintenance of high-quality instrument calibration, data acquisition/analysis, interpretation, and reporting of results (Licata et al., 2018). Therefore, exploring novel and effective screening diagnostic biomarkers that require minimal technical investment is crucial for the early screening and timely treatment of OP.

Maintenance of normal bone mass relies on a dynamic balance between bone resorption and formation. Emerging evidence has demonstrated that disruption of the balance, especially overactive osteoclast-induced bone resorption, predominates the progression of OP(Yao et al., 2017; Chen et al., 2020). N6-methyladenosine (m6A) modification is the most abundant internal modification in eukaryotic cells, affecting mRNA metabolism and various biological processes, including bone metabolic processes (Wei et al., 2017). m6A modification can be catalyzed by methyltransferase complexes, including METTL3, METTL14, WTAP, METTL16, RBM15, RBM15B, CBLL1, and ZC3H13, which can be removed by the demethylases ALKBH5 and FTO. Simultaneously, a variety of proteins that specifically recognize m6A sites have been found, including YTH family proteins (YTHDF1-3, YTHDC1-2) and ribonucleoproteins (HNRNPC), which can recognize m6A modification to regulate mRNA fates (Wu et al., 2018b). Increasing evidence has demonstrated the roles of m6A modification in diverse cancers by influencing their proliferation, migration, and invasion (An and Duan, 2022). Recently, the association between m6A modification and OP has also attracted the attention of some researchers. METTL3 is the most studied molecule and has different effects in different cell lines. In BMSCs, METTL3 functions as an inhibitor in OP to promote osteogenic differentiation and enhance bone formation by activating the PI3K-Akt signaling pathway or the PTH/Pth1r signaling axis (Wu et al., 2018a; Tian et al., 2019). However, another study reported that METTL3 could regulate osteoclast differentiation by increasing the bone resorption ability in RAW 264.7 cells, which may contribute to OP (Li D. et al., 2020). In addition, several studies have reported that FTO might be a new candidate for OP, which acts as an activator in OP, and its single nucleotide polymorphisms (SNPs) have a close relationship with BMD variation (DR, 1997; Guo et al., 2011; Li et al., 2019). Furthermore, YTHDF2 disrupts bone homeostasis by regulating osteoclast differentiation and inflammatory processes (Yu et al., 2019). The above findings demonstrate that m6A modification plays a vital role in OP. Nevertheless, gene signatures with diagnostic value for m6A modification in OP remain largely unstudied.

Various skeletal disorders have been found to be related to abnormalities in peripheral blood monocytes (PBMCs), which are widely accepted as the *in vivo* working cell model to study mechanisms in relation to OP(Zhou et al., 2015). PBMCs can migrate to the bone surface, differentiate into osteoclasts, and act as precursor cells of osteoclasts. Moreover, PBMCs produce essential cytokines for osteoclast differentiation, activation, and apoptosis (Kylmaoja et al., 2018). Recent advances in high-throughput technologies enable researchers to determine the molecular mechanisms and potential biomarkers of OP by isolating and analyzing the gene expression of PBMs. However, no such reports have systematically investigated the molecular mechanisms of m6A modification in OP using high-throughput data analysis.

In this study, we systematically analyzed the expression of m6A regulators mainly in PBMCs from different BMD samples, and the impact of m6A modification on bone metabolism microenvironment characteristics was also explored. Then, we performed consensus cluster analysis and identified two m6A modification patterns in low-BMD samples. In addition, we built a diagnostic model based on four key m6A regulators for distinguishing high- and low-BMD samples, and a regulatory network was then constructed to explore the possible regulatory mechanisms of m6A regulators in OP. Furthermore, we validated the altered m6A pattern of the four key regulators during RANKL- and/or MCSF induced osteoclast formation *in vitro*. Finally, an ovariectomized (OVX) mouse OP model was constructed to further validate the role of m6A modification in OP. Altogether, the present findings demonstrate that m6A regulators have a crucial impact on bone metabolism in OP, suggesting their future potential as diagnostic biomarkers of OP.

## 2 Materials and methods

### 2.1 Data collection and processing

We searched “osteoporosis” in the GEO and Array Express databases and retrieved datasets with a sample size greater than or equal to 80. Finally, two datasets were obtained, GSE56815 (https://www.ncbi.nlm.nih.gov/geo/query/acc.cgi?acc=GSE56815) and E-MEXP-1618 (https://www.ebi.ac.uk/arrayexpress/experiments/E-MEXP-1618/?query=osteoporosis&page=3&) The GSE56815 dataset contains the gene expression data of PBMCs from 80 Caucasian females, including 40 patients with high hip BMD (20 pre- and 20 postmenopausal) and 40 patients with low hip BMD (20 pre- and 20 postmenopausal), and this dataset served as the training dataset in the present study. The sample characteristics and RNA extraction protocol were well described in a previous study (Zhou et al., 2018). Moreover, the E-MEXP-1618 dataset served as the test dataset in this study, including 84 transiliac bone biopsies of postmenopausal females (50–86 years) with different BMDs. The detailed characteristics of the samples were presented in an early study (Reppe et al., 2010).

After downloading the two datasets, the probes were converted to gene symbols based on the corresponding annotation files. We only kept the probe with the largest numerical value when encountering probes corresponding to the same molecule. Then, we used the normalizeBetweenArrays function of the limma package to standardize the data, which was visualized with a box plot. Clustering of the samples was assessed through the principal component analysis (PCA) chart and the uniform manifold approximation and projection (UMAP) chart using the ggplot2 and umap packages.

### 2.2 Selection and expression analysis of m6A regulators

Sixteen widely recognized m6A regulators were selected from published literature, but the expression of two genes, METTL14 and ALKBH5, was not detected in the selected datasets, so the two genes were not included in this study. Therefore, 14 m6A regulators were involved in this study, namely, seven m6A writers, one m6A eraser, and six m6A readers (Table 1). The protein–protein interaction (PPI) network of these regulators was constructed using the STRING database (https://cn.string-db.org), and the expression correlations among the 14 m6A regulators in all samples were calculated by Spearman correlation analysis. To compare the expression differences of these m6A regulators between the high- and low-BMD samples, we used the limma package, and the results were visualized with a heatmap and box plot. Because the sample size was limited (although still among the largest of such studies in this field), we used a *p*-value <0.05 as the threshold for nominally significant differential expression.

**TABLE 1:** The description of 14 m6A RNA methylation regulators from the Ensembl database.

Gene	Ensembl	Type	Gene	Ensembl	Type
METTL3	ENSG00000165819	Writers	FTO	ENSG00000140718	Erasers
METTL16	ENSG00000127804	Writers	YTHDF1	ENSG00000149658	Readers
WTAP	ENSG00000146457	Writers	YTHDF2	ENSG00000198492	Readers
RBM15	ENSG00000162775	Writers	YTHDF3	ENSG00000185728	Readers
RBM15B	ENSG00000259956	Writers	YTHDC1	ENSG00000083896	Readers
CBLL1	ENSG00000105879	Writers	YTHDC2	ENSG00000047188	Readers
ZC3H13	ENSG00000123200	Writers	HNRNPC	ENSG00000092199	Readers

### 2.3 Analysis of the characteristics of the bone metabolic microenvironment

The bone metabolism-related gene sets were obtained from the GSEA database (http://www.gsea-msigbd.org/gsea/index.jsp) and were related to bone formation and bone resorption, such as bone remodeling, ossification, and multiple cellular processes of osteoclasts and osteoblasts (Supplementary Table S1). Single-sample gene set enrichment analysis (ssGSEA) was then used to calculate an enrichment score for each gene set in every sample, and we finally obtained the enrichment score matrix using the R package GSVA. The limma package was used to assess the changes in the abundance and activity of these gene sets in the high- and low-BMD samples, and the results are shown in a box plot. In addition, the relationship between the m6A regulators and these gene sets was evaluated by Spearman correlation analysis.

### 2.4 Identification of m6A modification patterns

To further explore the diverse m6A modification patterns in OP, unsupervised clustering analysis was employed to classify the low-BMD samples into different subtypes based on the expression of the 14 m6A regulators using the ConsensusClusterPlus package. Different modification patterns were verified by PCA using the ggplot2 package. Then, the distribution characteristics of m6A regulators and bone metabolism-related gene sets among the different subgroups were also compared using the limma package.

### 2.5 Construction of a diagnostic model based on the key m6A regulators

All 14 m6A regulators were used to perform univariate logistic regression, and the differentially expressed m6A regulators were included in multivariate logistic regression to further identify the key m6A regulators in OP. Then, these key genes serving as variables were used to construct the diagnostic model and calculate the risk score of each sample. Next, the median risk score was used as the cutoff, and the samples with a risk score higher than the median score were divided into the high-risk subgroup, whereas the samples with a risk score lower than the median were divided into the low-risk subgroup. The result was visualized with the risk factor graph using the ggplot2 package. Furthermore, the sensitivity and specificity of the model in the training and test datasets were determined by the ROC curve using the pROC package.

### 2.6 Creation of a network of m6A regulators-m6A target genes

All the targets of these key m6A regulators were screened from M6A2Target (http://m6a2target. canceromics.org), a comprehensive database for target genes of m6A modification, including validated targets reported in the articles and potential targets based on high-throughput sequencing data analysis. Then, a Venn diagram was generated to reveal the common and unique target genes of these m6A regulators. The common target genes coregulated by these key m6A regulators were further analyzed. Their biological functions in Gene Ontology (GO) and KEGG pathway enrichment were annotated using the clusterProfiler package. Finally, the regulatory network of these key m6A regulator-m6A target genes was built using Cytoscape software (version 3.9.1).

### 2.7 Cell culture and osteoclast differentiation

RAW264.7 cells (a murine macrophage cell line) were cultured in growth medium containing Dulbecco’s modified Eagle’s medium (DMEM; Gibco, Paisley, United Kingdom) and 10% fetal bovine serum (FBS; Gibco) in a humidified 5% CO2 incubator at 37°C. For gene expression analysis and TRAP staining, RAW264.7 cells were seeded at 1.5×10^4^ cells/well in 24-well plates in differentiation medium consisting of growth medium and 10 ng/ml nuclear factor (NF)-κB (RANKL; R&D Systems, Minnesota, United States). The osteoclast differentiation medium was changed every 2 days to induce differentiation, and the cells were cultured for 4 days.

Bone marrow-derived macrophages (BMMs) were isolated from the tibiae and femurs of 6- to 8-week-old C57BL/6 mice (Vital River Laboratory, Beijing, China) by flushing the bone marrow cavity with α-MEM. Then, the cells were cultured in α-MEM containing 10% FBS overnight to separate the suspended cells. The suspended cells were then collected and cultured in α-MEM containing 10% FBS with 10 ng/ml RANKL and 30 ng/ml mouse macrophage colony-stimulating factor (MCSF; R&D Systems). The medium was changed every 2 days to induce differentiation, and the cells were incubated at 37°C with 5% CO2 for 4 days. The experiment was approved by the Biomedical Ethics Committee of Peking University (issue number: LA2020199).

### 2.8 OVX model construction

Ten healthy female C57BL/6 mice aged 8 weeks (25–30 g) were randomly divided into two groups (*n* = 5 per group): the sham operation group and the OVX group. Ovaries were surgically removed on both sides after anesthesia, and then the wound was sutured. Eight weeks after surgery, blood samples were collected by eyeball plucking, and then PBMCs were isolated from blood samples using a mouse peripheral blood monocyte isolation kit according to the manufacturer’s protocols (Solarbio, Beijing, China). Briefly, 0.75–1 ml peripheral blood samples were collected from a 16-week-old mouse and diluted with an equal volume of phosphate buffered saline (PBS). Then, the white mononuclear cell layer was collected after density gradient centrifugation and washed with PBS three times followed by centrifugation at 250 *g* at room temperature for 10 min to obtain the mononuclear cell precipitate. Finally, we purified the cells by the differential adherent method. Cell precipitation was resuspended in 10% FBS DMEM and seed on a 24-well plate. Two to 4 hours after incubation, the inadherent cells were washed away, and the remaining monocytes were used for RNA extraction.

### 2.9 Tartrate-resistant acid phosphatase staining and osteoclasts counting

All culture media were pipetted out, and samples were washed with PBS three times and then fixed with 4% paraformaldehyde for 15 min at room temperature. Next, the cells were stained with a TRAP Kit (Sigma‒Aldrich Merck, Darmstadt, Germany) according to the manufacturer’s protocol for 40 min at 37°C in the dark. The cells were imaged using light microscopy (BX51, Olympus, Japan), and TRAP-positive cells were quantified as osteoclasts. This experiment was independently repeated three times.

### 2.10 Hematoxylin and eosin staining

HE staining of mouse femurs was used to detect bone destruction in OVX and sham mice. Femurs were dissected and fixed in 4% paraformaldehyde for 24 h, decalcified in 14% ethylene diamine tetraacetic acid (EDTA) at 37°C for 20 days, and then embedded into paraffin for sectioning. Bone sections were stained with HE (Beyotime Biotechnology, Shanghai, China) according to a standard protocol to quantify the surface area of bone and adipose tissues.

### 2.11 m6A quantification

Total m6A content was detected by a m6A RNA methylation assay kit (Abcam, Cambridge, United Kingdom) following the manufacture’s protocol. Briefly, total RNA samples of 200 ng for each group were administered with the solution containing the anti-m6A antibody. The m6A levels were quantified by using the colorimetric analysis *via* absorbance at 450 nm.

### 2.12 Real-time PCR

Total RNA was extracted with TRIzol reagent (Invitrogen, CA, United States) and obtained through chloroform isolation and isopropanol precipitation. Then, cDNA was generated *via* reverse transcription using a reverse transcription kit (Thermo Scientific, MA, United States). Next, the cDNA was amplified by a SYBR Kit (Roche Applied Science, IN, United States) on the ABI 7500 Sequencing Detection System (Applied Biosystems, CA, United States). RPS18 was used as a housekeeping gene, and the primer sequences used in this process are shown in Table 2.

**TABLE 2:** Primer pairs used in the real-time PCR

Genes	Forward primer	Reverse primer
METTL16	GAC​AAA​CCA​CCT​GAC​TTC​GCA	TCT​GAC​TGC​TTC​GGG​GTC​TT
FTO	TTC​ATG​CTG​GAT​GAC​CTC​AAT​G	GCC​AAC​TGA​CAG​CGT​TCT​AAG
CBLL1	GCG​AGC​CGA​ATC​ATG​GAT​CA	CTT​CTT​CAT​CAC​CTT​GCG​GG
YTHDF2	GAG​CAG​AGA​CCA​AAA​GGT​CAA​G	CTG​TGG​GCT​CAA​GTA​AGG​TTC
RPS18	TTC​CAG​CAC​ATT​TTG​CGA​GTA	CAC​GCC​CTT​AAT​GGC​AGT​GAT

### 2.13 Western blotting

The total protein was extracted using a RIPA kit (Huaxing Bio, Beijing, China), and then the protein concentration was quantified using a bicinchoninic acid (BCA) kit (Thermo Fisher). Protein samples (25 ug) were separated on electrophoresed in polyacrylamide gels and transferred onto polyvinylidene difluoride membranes (Millipore, MA, United States). After blocking in 5% skimmed milk at room temperature for 1 h, membranes were incubated with primary antibodies against FTO (Proteintech, Wuhan, China), METTL16 (Proteintech), YTHDF2 (Abcam), CBLL1 (Proteintech), and GAPDH (Huaxing bio) at 4°C overnight. The membranes were incubated with HRP-conjugated secondary antibodies (Huaxing Bio) for 1 h and visualized by an enhanced chemiluminescence blotting kit (Cwbiotech, Jiangsu, China). The intensities of the bands were quantified using Quantity One software (Bio-Rad, CA, United States). GAPDH was used as the internal control.

### 2.14 Statistical analysis

All the gene expression data from public datasets used in this study were processed using R software (version 3.6.3). For the gene expression data from public datasets, correlation analysis between these m6A regulators and the bone metabolism-related gene sets was conducted using the Spearman method. The limma R package was used to analyze these parameters between different groups. The m6A modification patterns were identified by unsupervised clustering analysis using the ConsensusClusterPlus package. Univariate and multivariate logistic regression analyses were applied to reduce the non-significant regulators, and the results were visualized using the forestplot package. The prediction efficiency of the diagnostic model was assessed by the ROC curve using the pROC package. The data from the experimental verification are presented as the mean ± standard deviation, and the comparison between two groups was performed using the two-tailed Student’s t test. All comparisons are presented as *p* values, and a *p*-value < 0.05 was considered statistically significant. Significant differences were considered at *p* < 0.05 *, *p* < 0.01 **, and *p* < 0.001 ***.

## 3 Results

### 3.1 Expression of m6A regulators in the high- and low-BMD groups

**FIGURE 1:**
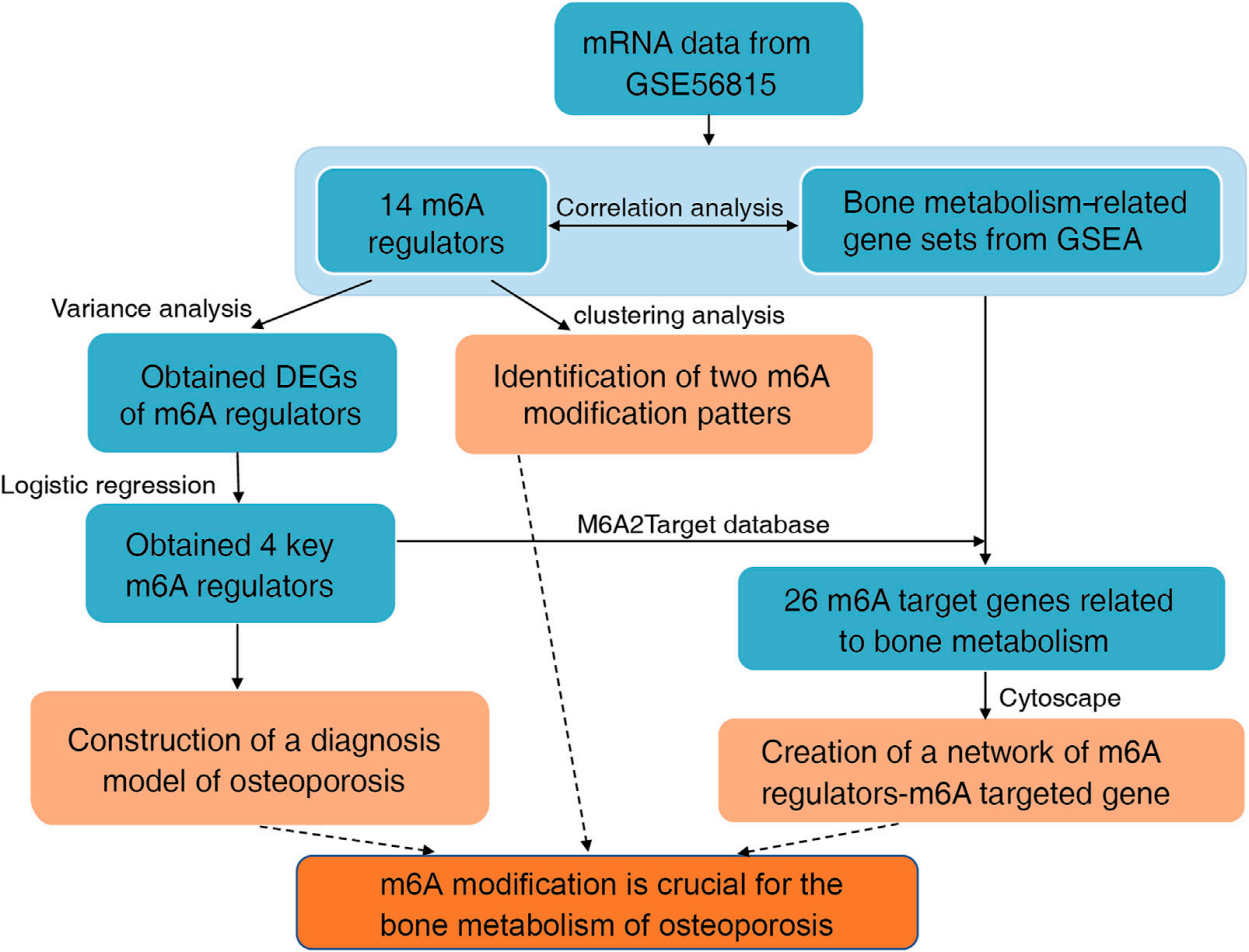
Flowchart and analysis strategy used in this study.

**FIGURE 2:**
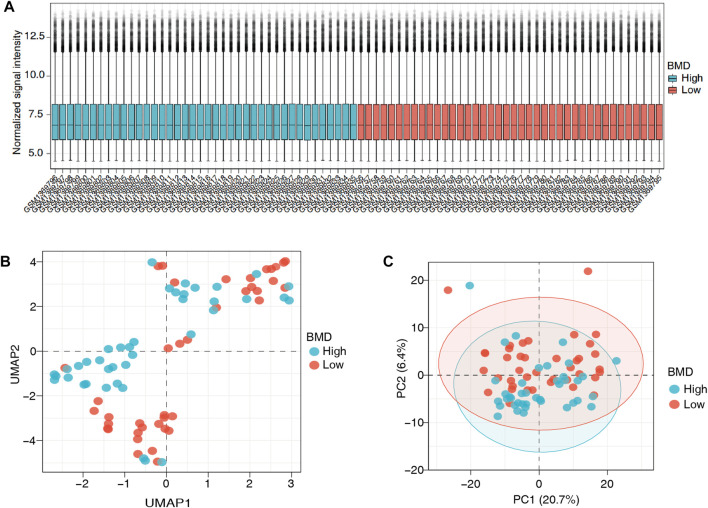
Standardization of gene expression. **(A)** Box plots of the gene expression data after normalization. **(B)** The uniform manifold approximation and projection (UMAP) plot and **(C)** principal component analysis (PCA) plot show the differences in gene expression between the two groups. The blue points represent the high bone mineral density (BMD) data, and the red points represent the low BMD data.

**FIGURE 3:**
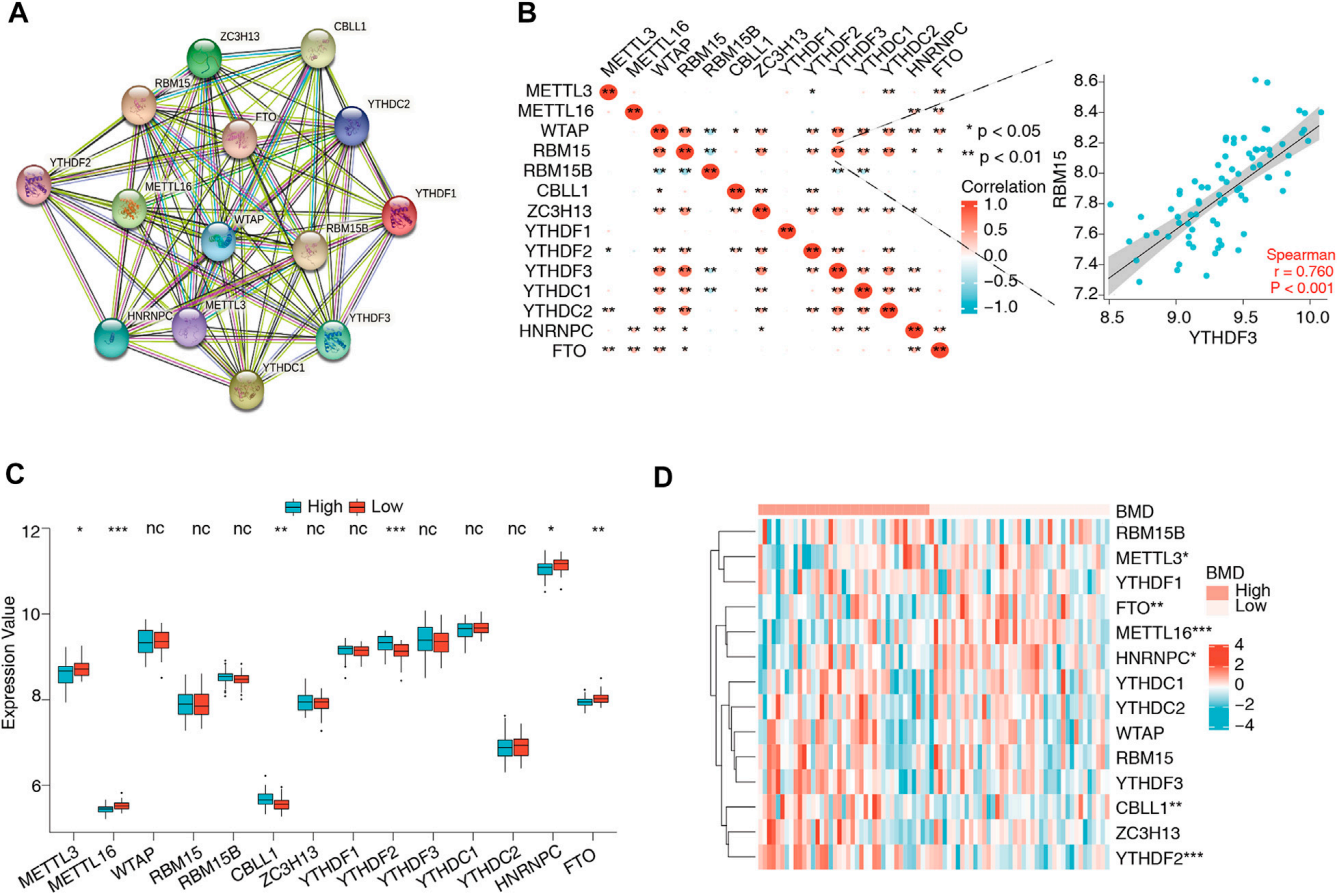
Expression and correlation of 14 m6A regulators in osteoporosis. **(A)** Protein–protein interactions of 14 m6A regulators. **(B)** Expression correlations of the 14 m6A regulators in all samples. The depth of the color block represents the level of the correlation coefficient, and * denotes the significance of the statistical analysis. The most correlated gene pair was *YTHDF2* and *RBM15*, the expression status of which is presented in the scatter plot in the right panel. **(C,D)** The box plot and heatmap plot show the summary of 14 m6A regulators between the high- and low-BMD groups, and six m6A regulators (*METTL3*, *METTL16*, *CBLL1*, *FTO*, *YTHDF2*, and *HNRNPC*) were significantly dysregulated.

The flowchart and analysis strategy used in the present study are shown in Figure 1. Before further analysis, the RNA expression data of GSE56815 were normalized (Figure 2A). UMAP and PCA plots were generated to reduce the dimensionality of the data and show the diverse gene expression patterns between the high- and low-BMD samples (Figures 2B,C). To explore the m6A modification patterns between the two groups, we thoroughly screened the complete gene expression profiles. There were 14 vital m6A regulators involved in the study, and their correlations were assessed at the protein and transcriptome levels. The PPI network was built on the STRING database and showed close direct physical interactions and indirect functional correlations between these m6A regulators (Figure 3A). Then, the correlation analysis revealed their strong relationship at the RNA level; notably, *YTHDF3* and *RBM15* were the most correlated genes, suggesting that they might work as a unit to act on OP (Figure 3B, Supplementary Table S2). Further variation analysis was performed to examine the expression differences in the 14 m6A regulators in the different groups (Figures 3C,D, Supplementary Table S3). Among these differentially expressed genes, four m6A regulators (*METTL3*, *METTL16*, *HNRNPC*, and *FTO*) were upregulated, and two m6A regulators (*CBLL1* and *YTHDF2*) were downregulated.

### 3.2 Correlations between m6A regulators and the bone metabolism microenvironment

**FIGURE 4:**
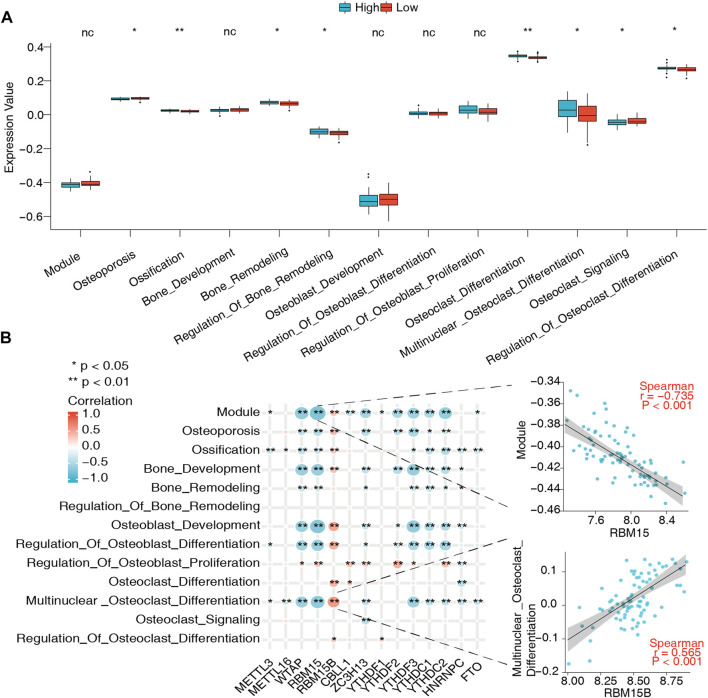
Relationship between bone metabolism-related gene sets and m6A regulators. **(A)** Differences in abundance and activity of bone metabolism-related gene sets in the high- and low-BMD groups. **(B)** Expression correlations of these bone metabolism-related gene sets and m6A regulators in all samples. Significantly, the *RBM15*-module pair was the most negatively correlated, the expression status of which is presented in the scatter plot in the upper right panel, while the *RBM15B*-multinuclear osteoclast differentiation pair was the most positively correlated with expression status presented in the scatter plot in the lower right panel.

As mentioned above, metabolic alterations in bone tissues contribute to BMD changes and OP occurrence. To probe their association with m6A regulators and the bone metabolism microenvironment, 13 bone metabolism-related gene sets were obtained from the GSEA database, and ssGSEA was used to calculate the relative enrichment score of each bone metabolism-related gene set in every sample. The results of the variation analysis are shown in Figure 4A; eight of the 13 bone metabolism-related gene sets were significantly dysregulated in low-BMD samples compared to high-BMD samples, illustrating the disturbance of the bone metabolic microenvironment in OP (Supplementary Table S4). Then, the correlations of m6A regulators with bone metabolism-related gene sets were explored. The results showed that they had a very close relationship, in which the *RBM15*-module pair was most negatively correlated (r = -0.735), while the *RBM15B*-multinuclear osteoclast pair was most positively correlated (r = 0.565) (Figure 4B, Supplementary Table S5).

### 3.3 Identification of two distinct m6A methylation patterns

**FIGURE 5:**
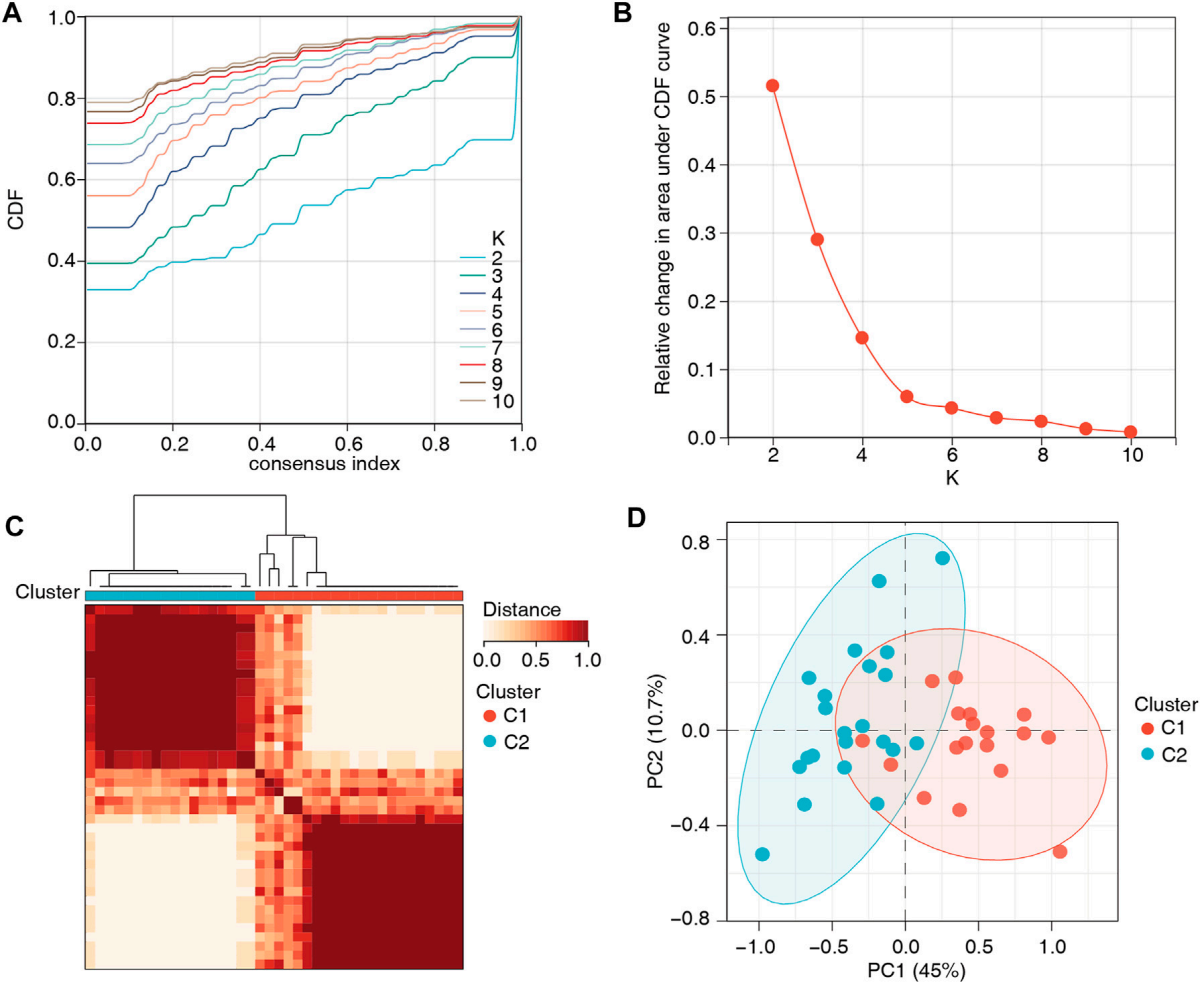
Unsupervised clustering analysis based on the 14 m6A regulators. **(A,B)** Consensus clustering cumulative distribution function (CDF) and the relative area under the CDF curve for k = 2–10. According to the recommendations for selecting the number of clusters, the number of clusters with the highest average consistency was k = 2. **(C)** The heatmap shows the consensus matrix for the optimal k = 2. **(D)** The PCA plot confirmed the striking difference between the two m6A modification patterns.

**FIGURE 6:**
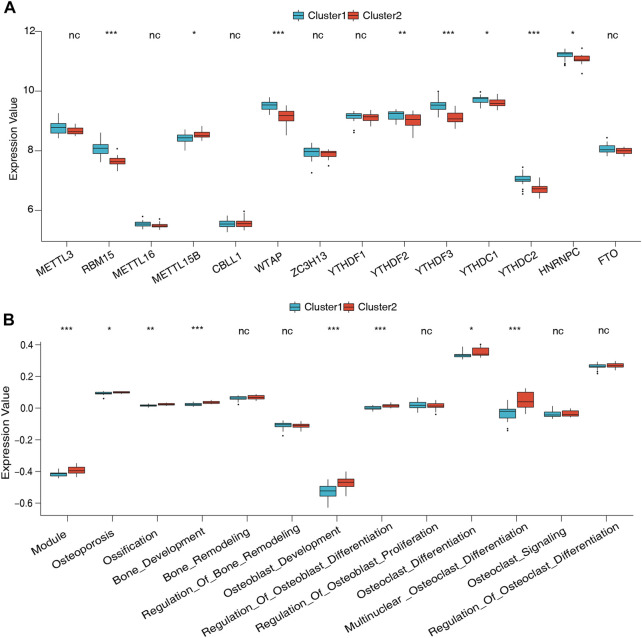
Expression of the m6A regulators and bone metabolism characteristics between the two m6A modification patterns. **(A)** Expression differences of 14 m6A regulators in the two m6A modification patterns. **(B)** Differences in the abundance and activity of bone metabolism-related gene sets in the two m6A modification patterns.

To further understand the role of m6A regulators in low BMD, unsupervised clustering analysis based on the 14 m6A regulators was performed and divided the low-BMD samples into two distinct m6A modification patterns, including 22 samples in cluster 1 and 18 samples in cluster 2 (Figures 5A–C, Supplementary Table S6). The PCA results confirmed that these m6A regulators could differentiate the two clusters in low-BMD samples (Figure 5D). Subsequently, we explored the expression of m6A regulators and bone metabolism-related gene sets between the two clusters. The variance analysis revealed that eight of 14 m6A regulators had a significant expression difference, validating the existence of diverse expression patterns mediated by m6A methylation modification in low-BMD samples (Figure 6A, Supplementary Table S7). Likewise, eight of 13 bone metabolism-related gene sets showed significant changes between the two clusters, and interestingly, we found that all these dysregulated gene sets were upregulated in cluster 2 compared to cluster 1, suggesting that cluster 2 might have more active bone metabolism characteristics (Figure 6B, Supplementary Table S8).

### 3.4 Construction and validation of a diagnostic model of OP

**FIGURE 7:**
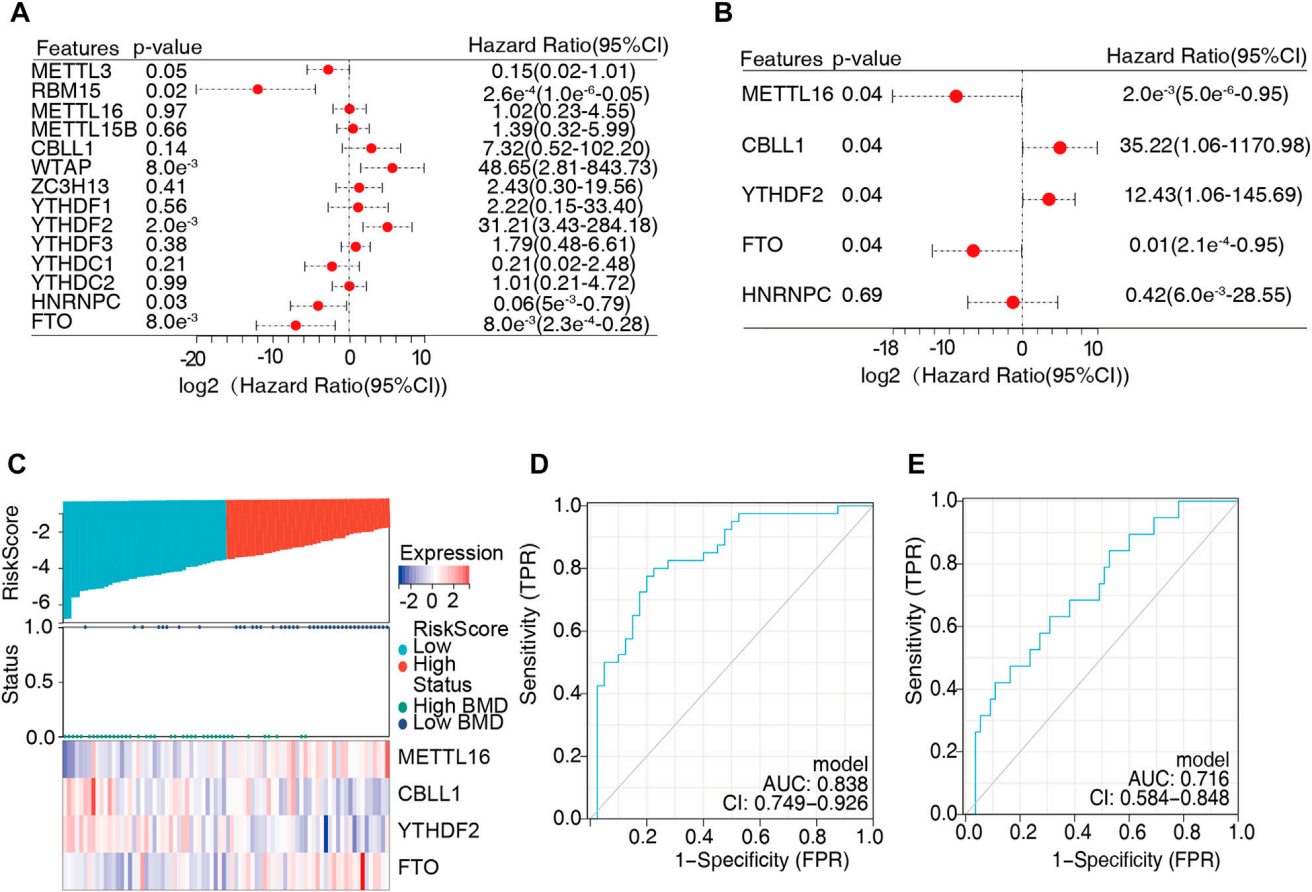
Construction of a diagnostic model of osteoporosis. **(A)** Univariate logistic regression analysis was conducted to identify the critical m6A regulators, indicating that five m6A regulators were significant for osteoporosis (*METTL16*, *CBLL1*, *YTHDF2*, *HNRNPC*, and *FTO*). **(B)** Multivariate logistic regression was employed to identify the independent modules, and four vital m6A regulators were obtained for the diagnostic model (*METTL16*, *CBLL1*, *YTHDF2*, and *FTO*). **(C)** The risk score was calculated based on the expression of the four vital m6A regulators, and the median risk score (–0.366) was used as the cutoff point. All samples were divided into two groups: the high-risk group and the low-risk group. **(D,E)** The sensitivity and specificity of the diagnostic model in the training dataset **(D)** and test dataset **(E)** were determined by receiver operating characteristic (ROC) curves, and the area under the curve was calculated.

The above findings indicated that m6A regulators were closely associated with bone metabolism-related gene sets and played an essential role in BMD and OP. Univariate logistic regression analysis was conducted to determine the differentially expressed genes, and five m6A regulators were found to be significantly correlated with BMD (Figure 7A, Supplementary Table S9). Then, we employed multivariate logistic regression to further reduce the unimportant regulators, and four key regulators were identified, namely, *METTL16*, *CBLL1*, *YTHDF2*, and *FTO* (Figure 7B, Supplementary Table S10). Next, these four key m6A regulators serving as variables were used to calculate the risk score of each sample and construct a diagnostic model of OP. The risk scores of the samples were determined (Supplementary Table S11), and the median risk score (-0.366) was used as the cutoff point to divide all the samples into two groups, namely, the high-risk group and the low-risk group. The high-risk and low-risk groups corresponded well to the low- and high-BMD groups, respectively (Figure 7C). In the diagnostic model, *CBLL1* and *YTHDF2* were protective factors, and their expression showed a downward trend with increasing risk score. *METTL16* and *FTO* were risk factors, and their expression showed an upward trend with increasing risk score. Furthermore, the ROC curve demonstrated that the expression values of the four key m6A regulators had moderate diagnostic accuracy (Figure 7D). The same result was also obtained for the test dataset (Figure 7E).

### 3.5 Creation of a BMD-related m6A regulator-m6A target gene regulatory network

**FIGURE 8:**
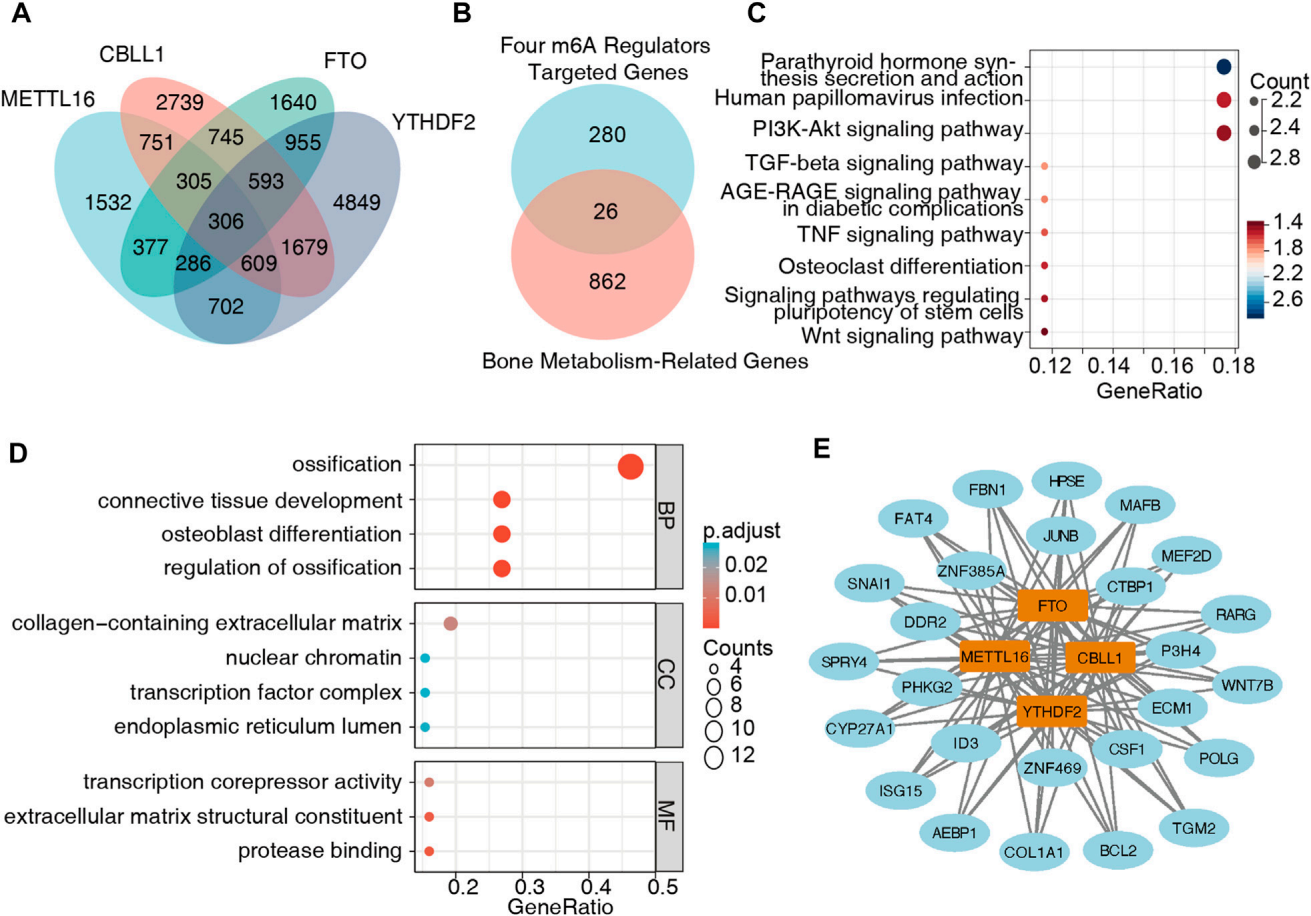
Creation of a regulatory network of m6A regulators-m6A target genes. **(A)** m6A target genes were obtained from M6A2Target, and 306 genes were potentially coregulated with the four m6A regulators. **(B)** Twenty-six m6A target genes were also closely related to bone metabolism. **(C)** KEGG pathway analysis showed that these 26 genes were mainly enriched in parathyroid hormone synthesis, secretion, action, human papillomavirus infection, and the P13K-AKT signaling pathway. **(D)** The GO analysis indicated that the biological processes of these genes were mainly enriched in ossification, regulation of ossification, connective tissue development, and osteoblast differentiation. **(E)** A regulatory network was built with four hub m6A regulators and 26 m6A target genes.

We obtained 4,868 METTL16 targets, 7,727 CBLL1 targets, 5,207 FTO targets, and 9979 YTHDF2 targets from M6A2Target, of which 306 genes were potentially coregulated with the four key m6A regulators (Figure 8A, Supplementary Table S12). Furthermore, these 306 targets were intersected with genes in the bone metabolism-related gene sets, and 26 target genes were finally obtained (Figure 8B, Supplementary Table S13). The KEGG pathway analysis showed that these genes were mainly enriched in parathyroid hormone synthesis, secretion, action, human papillomavirus infection, and the P13K-AKT signaling pathway, suggesting that these pathways might be closely related to BMD and OP (Figure 8C). The GO analysis indicated that the biological processes of these genes were mainly enriched in ossification, regulation of ossification, connective tissue development, and osteoblast differentiation, which were primarily related to bone metabolism (Figure 8D, Supplementary Table S14). Then, we used Cytoscape software and created a regulatory network composed of the four hub m6A regulators and the 26 m6A target bone metabolism-related genes (Figure 8E).

### 3.6 Validation of the expression of the key m6A regulators *in vitro* and *in vivo*

**FIGURE 9:**
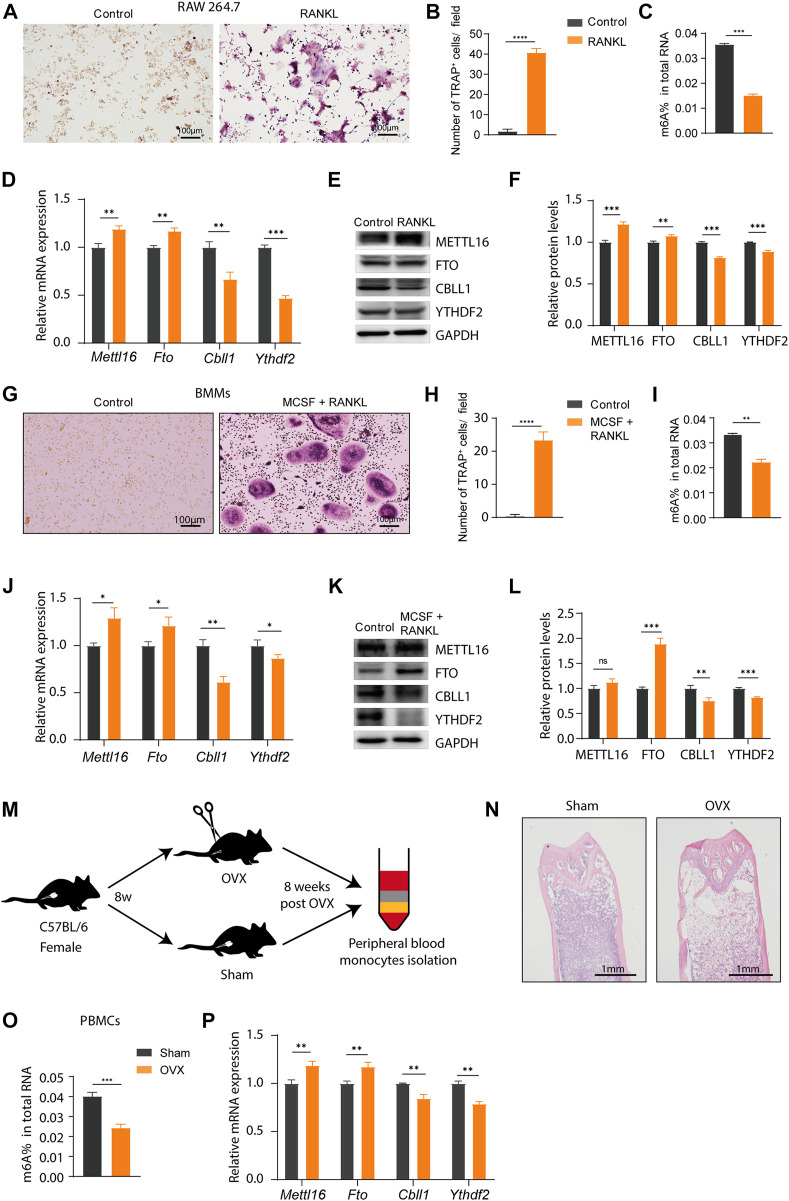
Validation of the expression of the key m6A regulators *in vitro* and *in vivo*. **(A,B)** Tartrate-resistant acid phosphatase (TRAP) staining and TRAP^+^ multinuclear cells counting of RAW 264.7 cells with or without nuclear factor (NF)-κB (RANKL) stimulation. Scale bar, 100 μm. **(C)** The m6A level in total RNA isolated from RAW264.7 cells during the osteoclast differentiation. **(D)** The expression of *Mettl16*, *Fto*, *Cbll1*, and *Ythdf2* in RAW264.7 cells was detected by real-time PCR after cultured with RANKL for 4 days **(E,F)** Western blotting and quantification of METTL16, FTO, CBLL1 and YTHDF2 in RAW264.7 cells after cultured in RANKL. **(G,H)** TRAP staining and TRAP^+^ multinuclear cells counting of bone marrow-derived macrophages (BMMs) with or without RANKL and macrophage colony-stimulating factor (MCSF) stimulation. Scale bar, 100 μm. **(I)** The m6A level in total RNA isolated from BMMs during the osteoclast differentiation. **(J)** The expression of *Mettl16*, *Fto*, *Cbll1*, and *Ythdf2* in BMMs was detected by real-time PCR after cultured with RANKL and MCSF for 4 days **(K,L)** Western blotting and quantification of METTL16, FTO, CBLL1, and YTHDF2 in BMMs after cultured in RANKL and MCSF. **(M)** A schematic diagram shows how peripheral blood monocytes (PBMCs) were obtained from the ovariectomized (OVX) and sham mice. **(N)** Representative images of Hematoxylin and eosin (HE) staining of mouse femurs showing the reduction of bone formation in the OVX mice relative to the sham-control counterparts. **(O)** The m6A level in total RNA isolated from PBMCs of the OVX and sham mice. **(P)** The mRNA expression level of *Mettl16*, *Fto*, *Cbll1*, and *Ythdf2* in PBMCs of the OVX and sham groups. Compared with the sham group.

To identify the reliability of the results based on bioinformatics analysis, we examined the expression of the four key m6A regulators (METTL16, CBLL1, YTHDF2, and FTO) *in vitro* and *in vivo*. RAW 264.7 cells, which are a classic cell line model for osteoclast and OP studies *in vitro*, were used in this study. RANKL treatment induced intense osteoclast differentiation of RAW264.7 cells (Figure 9A, Supplementary Figure S1A). Compared to control cells, a significantly elevated number of TRAP^+^ multinuclear osteoclasts formed upon RANKL stimulation for 4 days, indicating that the osteoclast induction model *in vitro* was successfully constructed (Figure 9B). We quantified the m6A content in total RNA by ELISA assays, and the m6A content was significantly decreased during osteoclast differentiation (Figure 9C). The expression patterns of METTL16, FTO, CBLL1, and YTHDF2 at the RNA and protein levels were examined in RAW264.7 cells, and the results showed downregulated expression of CBLL1 and YTHDF2 and upregulated expression of METTL16 and FTO during osteoclast differentiation (Figures 9D–F). Likewise, osteoclast differentiation induced from mouse BMMs were used for further validation. The number of TRAP^+^ multinuclear osteoclasts significantly increased upon RANKL- and MCSF stimulation. (Figures 9G,H, Supplementary Figure S1B). Next, we examined the total m6A level and the expression of four key genes in BMMs during osteoclast differentiation. The results were the same as those in RAW264.7 cells, except METTL16 at the protein level (Figures 9I–L). Finally, an OVX mouse model was constructed to represent the OP patients, and a schematic diagram was drawn to show how we obtained the PBMCs from mice (Figure 9M). Bone destruction was indicated by HE staining, and the bone mass was significantly decreased in OVX mice, which suggested that OP model was successfully constructed. (Figure 9N). We obtained the same total m6A level and mRNA expression data of these four key m6A regulators in PBMCs from the OVX model (Figures 9O, P). These results were consistent with our integrated analysis, indicating that the four key m6A regulators might be used as biomarkers of OP. However, the exact regulatory mechanism requires further study.

## 4 Discussion

OP, characterized by reduced BMD, is a widespread disease with a high prevalence in older women (Camacho et al., 2020). Abnormal bone metabolism, including enhanced bone resorption and diminished bone formation related to low sex hormones, is the primary pathological mechanism of OP in older adults (Awasthi et al., 2018). m6A RNA methylation is the most common epigenetic modification and is confirmed to be involved in almost every aspect of metabolism (Wei et al., 2017; Wu et al., 2018b). Studies have found that some m6A regulators, such as METTL3, FTO, and YTHDF2, play an essential role in bone metabolism by affecting the differentiation and proliferation of bone-related cells (Wu et al., 2018a; Li et al., 2019; Yu et al., 2019). However, an integrated bioinformatics analysis of various m6A regulators and bone metabolism characteristics in OP has not been systematically researched, which may increase understanding of the molecular mechanisms of m6A-mediated OP and provide some evidence for subsequent treatment.

We first searched GEO datasets and downloaded GSE56815 data concerning the gene expression of PBMCs in pre- and postmenopausal females, including 40 high-BMD and 40 low-BMD samples. First, we found that many m6A regulators have strong protein interactions or expression correlations, suggesting that they may function as complexes. The expression of most m6A regulators was altered between the high-BMD and low-BMD samples, illustrating that m6A regulators may be involve in OP development. Next, to investigate the relationship between m6A regulators and bone metabolism, we obtained 13 bone metabolism-related gene sets from the GESA database. Osteoporosis and osteoclast signaling gene sets were upregulated in the low-BMD group, while ossification, bone remodeling, and osteoclast differentiation, among other gene sets, were downregulated, implying the disturbance of the bone metabolic microenvironment in OP. In addition, we found that these bone metabolism-related gene sets were closely associated with m6A regulators. *RBM15* was most negatively connected with Module. The module pathway represents the degree of bone mineralization, which determines BMD (Roschger et al., 2014). A previous study demonstrated that circ-CTNNB1 interacted with RBM15 and subsequently promoted the aerobic glycolysis process (Yang et al., 2022). Meanwhile, aerobic glycolysis is critical for osteoclastogenesis, and increased aerobic glycolysis may induce excessive bone resorption and lead to osteoporotic fractures (Li B. et al., 2020). *RBM15B* was most positively connected with multinuclear osteoclast differentiation, which accelerates bone absorption and then promotes the occurrence and development of OP, while no relevant studies have explored the role of RBM15B in multinuclear osteoclast differentiation, which needs to be further studied in the future. However, it has been reported that METTL3 can modulate Atp6v0d2 mRNA degradation and Traf6 mRNA nuclear export to regulate osteoclast differentiation and function (Li D. et al., 2020). These results suggested that m6A modification had an essential regulatory role in shaping different bone metabolic microenvironments in OP.

Unsupervised clustering analyses have been used in several studies based on gene signatures to help elucidate the underlying mechanism of the studied disease (Zhang et al., 2020; Shen et al., 2021a; Liu et al., 2021). A recent study employed this method to comprehensively evaluate the m6A modification patterns among 9,804 pancancer samples and identified three distinct m6A modification subtypes, which enhanced our understanding of the dysregulation of RNA methylation in tumor microenvironments (Shen et al., 2021b). We used 14 m6A signatures and developed two distinct m6A modification subgroups with different bone metabolism microenvironments in the low-BMD group. Compared with cluster 1, cluster 2 had more active bone metabolic activities. The unique characteristics of bone metabolism between the two clusters verified the feasibility of classifying the bone metabolic microenvironment by m6A regulators. Simultaneously, our findings aid a deeper understanding of the molecular mechanisms of OP and may be used as a basis for individualized choice of drug therapy (Marozik et al., 2019). Unsupervised clustering analyses have also been used in some clinical studies of OP. A study divided patients into nine subgroups with significant differences in clinical features, BMD distribution, and medical care costs. It quantified patients into three different fracture risk levels, which showed a better understanding of fracture risk phenotypes (Kruse et al., 2017).

We evaluated the role of m6A regulators in diagnosing OP or the BMD phenotype using univariate and multivariate logistic regression analyses, which are widely applied in diagnosing diseases such as periodontitis and appendicitis (Eddama et al., 2019; Zhang et al., 2021). Four key m6A regulators significantly associated with the BMD phenotype were chosen for the diagnostic model. In this model, patients with high *CBLL1* and *YTHDF2* expression had a low likelihood of decreased bone density. In contrast, patients with high expression of *METTL16* and *FTO* had an increased risk of OP. Subsequently, the risk score of all the samples was evaluated. The results showed that patients with low BMD had a higher risk score, suggesting their potential clinical value for the diagnosis of OP. Furthermore, the model’s predictive power was assessed by ROC analysis, which showed moderate accuracy. The same result was also obtained in the test dataset, which further verified the extrapolation of the results. The roles of FTO and YTHDF2 have been studied in OP. FTO promotes OP through demethylating Runx2 mRNA and inhibiting osteogenic differentiation (Wang et al., 2021). YTHDF2 might be involved in regulation of the lipopolysaccharide (LPS)-stimulated inflammatory reactions *via* regulating the stability of MAP2K4 and MAP4K4 mRNAs in RAW 264.7 cells (Yu et al., 2019). However, CBLL1 and METTL16 have mainly been studied in cancers and act as oncogenic markers to promote the development and progression of tumors (Hui et al., 2019; Su et al., 2022). Their role in OP has not been reviewed, which guides us to further explore their relevant roles in the OP field.

A gene regulatory network containing the four hub m6A regulators and 26 m6A target genes related to bone metabolism was constructed to further understand the role of m6A regulators in OP. The biological processes of these target genes were mainly enriched in ossification, implying their essential role in OP or BMD. In addition, KEGG analysis revealed that these genes primarily focused on parathyroid hormone synthesis, secretion, action, human papillomavirus infection, and the P13K-AKT signaling pathway. Parathyroid hormone has been reported to augment bone formation, particularly in trabecular and cortical bone, and has a central role in regulating extracellular fluid Ca ^++^ and phosphate (Pi) homeostasis (Goltzman, 2018). One study has showed that METTL3 reduces the translation efficiency of the bone marrow stem cell (BMSC) lineage allocator parathyroid hormone receptor 1 and disrupts parathyroid hormone-induced osteogenic and adipogenic responses to promote OP (Wu et al., 2018a). There is no related research on HPV infection and OP, but one study found higher mean alveolar bone loss in patients with HPV-positive tumors (Mine Tezal et al., 2009). The PI3K-AKT signaling pathway has been reported to be involved in various cellular processes, including BMSCs proliferation and osteoclast differentiation (Shen G. Y. et al., 2018). Conditional knockdown of METTL3 in BMSC suppressed PI3K-Akt signaling and limited the expression of bone formation-related genes to regulate osteogenic differentiation and alternative splicing of Vegfa in BMSC(Tian et al., 2019). These findings may provide a foundation for m6A modification in OP and imply a direction for the relationship between m6A regulators and bone metabolism-related genes in OP.

Finally, we verified the expression of the key m6A regulators *in vivo* and *in vitro* models of OP. Excessive osteoclast activity results in reduced bone mass and decreased bone strength in OP, hence, osteoclasts are considered therapeutic targets for bone-related diseases including OP. In the present study, we established RANKL- and/or MCSF-induced BMMs and RAW264.7 cells as osteoclast differentiation cell models (Kim et al., 2020). In addition, we constructed animal models of OP to further investigate our results, and the OVX model is the most utilized approach in such studies (Fu et al., 2020). We first quantified m6A contents and found that the total m6A levels were significantly decreased in osteoclast differentiation cells and OVX mice, which was consistent with the related research in OP (Yan et al., 2020). The expression of METTL16, CBLL1, YTHDF2, and FTO at the RNA and protein levels was consistent with our bioinformatics analysis results. However, interestingly, METTL16 and FTO, which exhibit opposing m6A catalytic abilities, were significantly upregulated in RAW264.7 cells, BMMs and PBMCs. The upregulation trend of FTO, the most important demethylase, was consistent with the decreasing m6A level and downregulated expression of CBLL1 and YTHDF2, while METTL16 exhibited a negative correlation with that. One possible explanation for the increased METTL16 might be that METTL16 could be compensating for the feedback of descending m6A modification induced by FTO, CBLL1 and YTHDF2 in RAW264.7 cells, BMMs and PBMCs. The phenomenon that these enzymes with opposite functions have the same expression trend is common in other m6A-related studies (Ma et al., 2017). The OVX mouse model is an excellent preclinical model. However, because a small amount of peripheral blood, approximately 0.75–1 ml for each mouse, can be obtained, the number of PMBCs is limited. These PMBCs could obtain approximately 1 ug of RNA, which met the experimental verification at the RNA level. However, those PMBCs were not enough for protein level verification, and the relevant validation needs to be carried out in other large animals or clinical trials in the future.

These findings further illustrated the impact of m6A regulators on the bone metabolic microenvironment of OP. However, there are still some limitations to our study. First, collecting blood samples from human patients is an invasive operation. Considering that our study is a preliminary exploratory study, it cannot benefit patients for the time being. Especially during the COVID-19 pandemic, due to the requirements of ethics and social management, we were unable to collect human samples, which are more credible than cell lines and mouse samples. Of course, if we can collect some blood samples during the operation of OP patients in the future, we will carry out corresponding experiments for further verification. In addition, the datasets on OP presently lack a more extensive sample study, so extrapolation of the above results may be limited due to the small sample size of our study. Finally, our study mainly focused on exploring the role of m6A modification in the diagnosis of OP, and we did not investigate the specific regulatory mechanism of m6A regulators in OP. Relevant studies have shown that FTO can regulate the occurrence and development of OP through the GDF11-FTO-Pparg axis, which can be used as a potential therapeutic target (Shen G. S. et al., 2018). Moreover, only a limited number of FTO inhibitors have been identified, yet their efficacy and safety are inconclusive. Notably, there are currently no m6A-based drugs developed for OP. Therefore, to address these limitations, we still have a long way to go.

## 5 Conclusion

In conclusion, we preliminarily explored the implications of m6A regulators in OP by identifying two m6A modification patterns and constructing a regulatory network of the m6A regulator-m6A target genes. In addition, we successfully identified four m6A regulators, namely, *METTL16*, *CBLL1*, *YTHDF2*, and *FTO*, as potential biomarkers for diagnosing OP and the expression of the four key m6A regulators was validated *in vitro* and *in vivo*. Taken together, our results revealed that m6A modification has essential roles in OP, which may imply a direction for us to further explore the specific mechanism of these m6A regulators in OP.

## Data Availability

The original contributions presented in the study are included in the article/Supplementary Material, further inquiries can be directed to the corresponding authors.
