# Supplementary material for: Identification and experimental validation of key m6A modification regulators as potential biomarkers of osteoporosis
Source: Front Genet. 2023 Jan 6;13:1072948. doi: 10.3389/fgene.2022.1072948 (PMC9852729; doi:10.3389/fgene.2022.1072948)
Supplement: Supplementary file 1 [file DataSheet1.pdf]

Supplementary Material

1. Supplementary Tables

Supplementary Table 1. The description of 13 bone metabolism-related gene sets from GSEA database.

Name of gene set	Number of genes	Description
Module	54	Bone remodeling.
Osteoporosis	285	Osteoporosis
Ossification	419	The formation of bone or of a bony substance, or the conversion of fibrous tissue or of cartilage into bone or a bony substance.
Bone_Development	218	The process whose specific outcome is the progression of bone over time, from its formation to the mature structure. Bone is the hard skeletal connective tissue consisting of both mineral and cellular components.
Bone_Remodeling	88	The continuous turnover of bone matrix and mineral that involves first, an increase in resorption (osteoclastic activity) and later, reactive bone formation (osteoblastic activity). The process of bone remodeling takes place in the adult skeleton at discrete foci. The process ensures the mechanical integrity of the skeleton throughout life and plays an important role in calcium homeostasis. An imbalance in the regulation of bone resorption and bone formation results in many of the metabolic bone diseases, such as osteoporosis.
Regulation_Of_Bone _Remodeling	49	Any process that modulates the frequency, rate or extent of bone remodeling, the processes of bone formation and resorption that combine to maintain skeletal integrity.
Osteoblast_Development	18	The process whose specific outcome is the progression of an osteoblast over time, from its formation to the mature structure. Osteoblast development does not include the steps involved in committing a cranial neural crest cell or an osteoprogenitor cell to an osteoblast fate. An osteoblast is a cell that gives rise to bone.
Regulation_Of_Osteoblast _Differentiation	135	Any process that modulates the frequency, rate or extent of osteoblast differentiation.

Regulation_Of_Osteoblast _Proliferation	28	Any process that modulates the frequency, rate or extent of osteoblast proliferation.
Osteoclast_Differentiation	96	The process in which a relatively unspecialized monocyte acquires the specialized features of an osteoclast. An osteoclast is a specialized phagocytic cell associated with the absorption and removal of the mineralized matrix of bone tissue.
Multinuclear _Osteoclast _Differentiation	8	The process in which a relatively unspecialized monocyte acquires the specialized features of a multinuclear osteoclast. An osteoclast is a specialized phagocytic cell associated with the absorption and removal of the mineralized matrix of bone tissue.
Osteoclast_Signaling	16	Osteoclast signaling
Regulation_Of_Osteoclast _Differentiation	64	Any process that modulates the frequency, rate or extent of osteoclast differentiation.

Supplementary Table 2. Correlation of the m6A regulators.

**Gene**	**Gene**	**Correlation**	***P* value**
METTL3	METTL16	0.215050487	0.055406365
METTL3	WTAP	-0.027906868	0.805894332
METTL3	RBM15	0.101606315	0.369817458
METTL3	RBM15B	-0.135783731	0.229773066
METTL3	CBLL1	0.000738401	0.994813377
METTL3	ZC3H13	-0.023406138	0.836725142
METTL3	YTHDF1	0.218918301	0.051059015
METTL3	YTHDF2	-0.264077966	0.017933191
METTL3	YTHDF3	0.013209173	0.907421102
METTL3	YTHDC1	0.203329837	0.070456889

METTL3	YTHDC2	0.296333195	0.007607679
METTL3	HNRNPC	0.173723475	0.123287842
METTL3	FTO	0.300038678	0.006851177
METTL16	WTAP	0.163830286	0.146470557
METTL16	RBM15	0.061415846	0.588379496
METTL16	RBM15B	-0.100468823	0.375227531
METTL16	CBLL1	-0.185700891	0.099107284
METTL16	ZC3H13	-0.063056728	0.578434075
METTL16	YTHDF1	0.018518519	0.870485545
METTL16	YTHDF2	-0.205883732	0.06692463
METTL16	YTHDF3	0.034646038	0.760291448
METTL16	YTHDC1	0.077168308	0.496272148
METTL16	YTHDC2	0.055414909	0.625393526
METTL16	HNRNPC	0.352273793	0.001352347
METTL16	FTO	0.390684427	0.000339798
WTAP	RBM15	0.720581341	4.86278E-14
WTAP	RBM15B	-0.438959212	4.63834E-05
WTAP	CBLL1	0.278082513	0.012504665
WTAP	ZC3H13	0.454617909	2.27283E-05
WTAP	YTHDF1	-0.028973277	0.798630528
WTAP	YTHDF2	0.381622128	0.000478004
WTAP	YTHDF3	0.68649789	2.07878E-12

WTAP	YTHDC1	0.534130333	3.35044E-07
WTAP	YTHDC2	0.556000938	8.59625E-08
WTAP	HNRNPC	0.336943272	0.002241766
WTAP	FTO	0.398748235	0.000248727
RBM15	RBM15B	-0.473863104	9.00394E-06
RBM15	CBLL1	0.214181903	0.056423101
RBM15	ZC3H13	0.561814346	5.88902E-08
RBM15	YTHDF1	0.158579466	0.16003247
RBM15	YTHDF2	0.42962963	6.98007E-05
RBM15	YTHDF3	0.760454759	4.44089E-16
RBM15	YTHDC1	0.495546179	2.96003E-06
RBM15	YTHDC2	0.636451008	2.22466E-10
RBM15	HNRNPC	0.249038912	0.025904666
RBM15	FTO	0.246215695	0.027695538
RBM15B	CBLL1	-0.004125645	0.97102699
RBM15B	ZC3H13	-0.158556024	0.160095033
RBM15B	YTHDF1	0.142170652	0.208391421
RBM15B	YTHDF2	-0.040342241	0.722366574
RBM15B	YTHDF3	-0.369737459	0.000736969
RBM15B	YTHDC1	-0.380661041	0.000495338
RBM15B	YTHDC2	-0.157477731	0.162992566
RBM15B	HNRNPC	-0.200070323	0.075181254

RBM15B	FTO	-0.146883186	0.193549241
CBLL1	ZC3H13	0.361978434	0.000969189
CBLL1	YTHDF1	0.132090952	0.242811143
CBLL1	YTHDF2	0.3201594	0.003789854
CBLL1	YTHDF3	0.145100797	0.199070711
CBLL1	YTHDC1	0.04556962	0.688139724
CBLL1	YTHDC2	0.186779184	0.097126882
CBLL1	HNRNPC	-0.179559306	0.110995778
CBLL1	FTO	0.073007929	0.519844471
ZC3H13	YTHDF1	0.155578997	0.168188711
ZC3H13	YTHDF2	0.479301453	6.86014E-06
ZC3H13	YTHDF3	0.585114862	1.19932E-08
ZC3H13	YTHDC1	0.322995781	0.003475093
ZC3H13	YTHDC2	0.391701828	0.000326825
ZC3H13	HNRNPC	0.223206751	0.046570432
ZC3H13	FTO	0.138221626	0.221437992
YTHDF1	YTHDF2	0.126277543	0.264353821
YTHDF1	YTHDF3	0.057430849	0.612849228
YTHDF1	YTHDC1	0.072151899	0.524763936
YTHDF1	YTHDC2	0.14254571	0.207181358
YTHDF1	HNRNPC	0.039029536	0.731051138
YTHDF1	FTO	0.149004624	0.187122271

YTHDF2	YTHDF3	0.413009845	0.000140442
YTHDF2	YTHDC1	0.208110642	0.063962582
YTHDF2	YTHDC2	0.367416784	0.000800459
YTHDF2	HNRNPC	-0.137623066	0.223464487
YTHDF2	FTO	-0.117101014	0.300919669
YTHDF3	YTHDC1	0.476699484	7.81788E-06
YTHDF3	YTHDC2	0.60222691	3.43306E-09
YTHDF3	HNRNPC	0.340506329	0.001997771
YTHDF3	FTO	0.213339272	0.057423962
YTHDC1	YTHDC2	0.520745429	7.35545E-07
YTHDC1	HNRNPC	0.31432255	0.004518631
YTHDC1	FTO	0.216468685	0.053778483
YTHDC2	HNRNPC	0.092194093	0.416008696
YTHDC2	FTO	0.196098196	0.081277692
HNRNPC	FTO	0.387965237	0.000376825

Supplementary Table 3. Expression diversity of the 14 m6A regulators between high- and low-BMD samples.

**Tag**	**FC**	**AveExpr**	**t**	***P*. Value**	**adj.*P*. Val**	**B**
METTL3	0.956664399	3.1160173	-2.10399153	0.038402917	0.22529069	-5.097285451
RBM15	1.012265013	2.97884305	0.435066405	0.664642499	0.86095308	-7.173799072
METTL16	0.951531156	2.45481228	-3.56476809	0.000606979	0.03468704	-1.316145203

RBM15B	1.023189249	3.08700362	1.49440069	0.138860103	0.41956655	-6.159256956
CBLL1	1.069048624	2.48889047	2.966305378	0.003934756	0.07725931	-3.058132193
WTAP	1.000138721	3.22341954	0.005926955	0.995285213	0.9987423	-7.268997356
ZC3H13	1.016418939	2.98700669	0.81610805	0.416772492	0.70361658	-6.934937593
YTHDF1	1.007536715	3.19193927	0.547178555	0.585723147	0.81654308	-7.118504019
YTHDF2	1.065820265	3.20101988	3.435202477	0.000926628	0.04013846	-1.713903935
YTHDF3	1.022698928	3.22636045	0.840026178	0.40330501	0.6927217	-6.915152865
YTHDC1	0.982217216	3.2695535	-1.25645401	0.212473383	0.51312566	-6.481471622
YTHDC2	0.999171272	2.78436279	-0.02752699	0.97810544	0.9919114	-7.268633445
HNRNPC	0.971940519	3.47109251	-2.21463343	0.029523805	0.19921479	-4.869506929
FTO	0.962290308	2.99759538	-2.88705344	0.004955405	0.08542457	-3.269807546

Supplementary Table 4. Diversity of bone metabolism-related gene sets between high- and low-BMD samples

**Tag**	**FC**	**AveExpr**	**t**	***P*. Value**	**adj.*P*. Val**	**B**
Osteoclast _Differentiation	1.02291462	0.341892516	3.356222106	0.001211415	0.015748399	-2.791576467
Ossification	1.011358488	0.022423376	3.104191867	0.002638918	0.01715297	-3.519853677
Regulation_Of _Bone _Remodeling	1.024292261	-0.104808248	2.432461202	0.017231149	0.073875956	-5.234348423
Osteoporosis	0.991099655	0.094405769	-2.23222644	0.028399336	0.073875956	-5.676615113
Regulation_Of	1.018328711	0.270386109	2.196338255	0.030966114	0.073875956	-5.752354935

_Osteoclast _Differentiation						
Bone_Remodeling	1.013682295	0.067874561	2.145967587	0.034909909	0.073875956	-5.856821396
Multinuclear _Osteoclast _Differentiation	1.074512201	0.018524576	2.09019833	0.039779361	0.073875956	-5.969961532
Osteoclast _Signaling	0.97743646	-0.04133151	-2.000069503	0.048889914	0.07944611	-6.147145848
Regulation_Of _Osteoblast _Proliferation	1.025212481	0.022300867	1.852973754	0.067577526	0.097611983	-6.421075511
Module	0.982729283	-0.40904398	-1.581589995	0.117693284	0.153001269	-6.875651296
Bone _Development	0.993922012	0.027567812	-1.096202286	0.276282816	0.326516056	-7.518211098
Regulation_Of _Osteoblast _Differentiation	1.004711193	0.007927953	0.666772757	0.506837923	0.549074416	-7.897248422
Osteoblast _Development	0.995307576	-0.501441631	-0.180239719	0.857420665	0.857420665	-8.105075735

Supplementary Table 5. Correlations between m6A regulators and bone metabolism-related gene sets.

Gene	Bone metabolism-related gene sets	Correlation	*P* value
METTL3	Module	-0.224251197	0.045528034
METTL3	Osteoporosis	-0.11394816	0.314210927

METTL3	Ossification	-0.3274398	0.003028676
METTL3	Bone_Development	-0.016291704	0.885947795
METTL3	Bone_Remodeling	-0.089041778	0.432194803
METTL3	Regulation_Of_Bone_Remodeling	0.04579258	0.68669313
METTL3	Osteoblast_Development	-0.174168859	0.122314469
METTL3	Regulation_Of_Osteoblast_Differentiation	-0.243226929	0.029704406
METTL3	Regulation_Of_Osteoblast_Proliferation	-0.184904975	0.100589234
METTL3	Osteoclast_Differentiation	-0.170113514	0.131397448
METTL3	Multinuclear _Osteoclast_Differentiation	-0.249942862	0.025352425
METTL3	Osteoclast_Signaling	-0.040647214	0.720353904
METTL3	Regulation_Of_Osteoclast_Differentiation	-0.103927004	0.358928309
METTL16	Module	-0.039568683	0.7274801
METTL16	Osteoporosis	0.204477262	0.06885178
METTL16	Ossification	-0.246952649	0.027218251
METTL16	Bone_Development	0.102461322	0.365782386
METTL16	Bone_Remodeling	-0.070839194	0.532352977
METTL16	Regulation_Of_Bone_Remodeling	-0.018706048	0.869185535
METTL16	Osteoblast_Development	-0.100515706	0.375003609
METTL16	Regulation_Of_Osteoblast_Differentiation	-0.027684013	0.807414396
METTL16	Regulation_Of_Osteoblast_Proliferation	-0.178996718	0.112137743
METTL16	Osteoclast_Differentiation	-0.205368026	0.067626132
METTL16	Multinuclear _Osteoclast_Differentiation	-0.306540084	0.005682853

METTL16	Osteoclast_Signaling	0.190436006	0.090640974
METTL16	Regulation_Of_Osteoclast_Differentiation	-0.091068917	0.421745419
RBM15	Module	-0.734903891	8.43769E-15
RBM15	Osteoporosis	-0.426136896	8.1094E-05
RBM15	Ossification	-0.327191749	0.003052172
RBM15	Bone_Development	-0.612822316	1.52382E-09
RBM15	Bone_Remodeling	-0.302906704	0.006311966
RBM15	Regulation_Of_Bone_Remodeling	0.006610408	0.953593015
RBM15	Osteoblast_Development	-0.708415377	1.9762E-13
RBM15	Regulation_Of_Osteoblast_Differentiation	-0.602133146	3.45737E-09
RBM15	Regulation_Of_Osteoblast_Proliferation	0.318471636	0.003988981
RBM15	Osteoclast_Differentiation	-0.16432255	0.145244754
RBM15	Multinuclear _Osteoclast_Differentiation	-0.652906704	5.26876E-11
RBM15	Osteoclast_Signaling	-0.17862166	0.112904086
RBM15	Regulation_Of_Osteoclast_Differentiation	-0.053187998	0.63937519
RBM15B	Module	0.321143929	0.003677831
RBM15B	Osteoporosis	0.351664323	0.001380462
RBM15B	Ossification	0.305625879	0.005835688
RBM15B	Bone_Development	0.349437412	0.001487738
RBM15B	Bone_Remodeling	0.183778715	0.102715788
RBM15B	Regulation_Of_Bone_Remodeling	-0.006680731	0.953099878
RBM15B	Osteoblast_Development	0.496952649	2.74665E-06

RBM15B	Regulation_Of_Osteoblast_Differentiation	0.457759025	1.96144E-05
RBM15B	Regulation_Of_Osteoblast_Proliferation	-0.065541491	0.563523215
RBM15B	Osteoclast_Differentiation	0.412025316	0.000146215
RBM15B	Multinuclear _Osteoclast_Differentiation	0.564744491	4.85342E-08
RBM15B	Osteoclast_Signaling	-0.065142991	0.565902302
RBM15B	Regulation_Of_Osteoclast_Differentiation	0.282372246	0.011157242
CBLL1	Module	-0.337083919	0.002231649
CBLL1	Osteoporosis	-0.157735584	0.162296175
CBLL1	Ossification	0.181340835	0.107438888
CBLL1	Bone_Development	-0.10424285	0.357461728
CBLL1	Bone_Remodeling	0.040600094	0.720664752
CBLL1	Regulation_Of_Bone_Remodeling	0.134716362	0.233490381
CBLL1	Osteoblast_Development	-0.026113455	0.818146901
CBLL1	Regulation_Of_Osteoblast_Differentiation	-0.034646038	0.760291448
CBLL1	Regulation_Of_Osteoblast_Proliferation	0.328715424	0.002910384
CBLL1	Osteoclast_Differentiation	0.283052039	0.010955759
CBLL1	Multinuclear _Osteoclast_Differentiation	-0.05443038	0.63155899
CBLL1	Osteoclast_Signaling	-0.126887014	0.26203656
CBLL1	Regulation_Of_Osteoclast_Differentiation	0.189428036	0.092393655
WTAP	Module	-0.644514768	1.11031E-10
WTAP	Osteoporosis	-0.362658228	0.000946463
WTAP	Ossification	-0.384575715	0.000428147

WTAP	Bone_Development	-0.605860291	2.6072E-09
WTAP	Bone_Remodeling	-0.331575246	0.002660082
WTAP	Regulation_Of_Bone_Remodeling	0.009774965	0.931421932
WTAP	Osteoblast_Development	-0.594655415	6.02517E-09
WTAP	Regulation_Of_Osteoblast_Differentiation	-0.548710736	1.3675E-07
WTAP	Regulation_Of_Osteoblast_Proliferation	0.234153774	0.036570118
WTAP	Osteoclast_Differentiation	-0.090365682	0.425353851
WTAP	Multinuclear _Osteoclast_Differentiation	-0.585067979	1.20332E-08
WTAP	Osteoclast_Signaling	-0.057102672	0.614883887
WTAP	Regulation_Of_Osteoclast_Differentiation	-0.022151899	0.845363045
ZC3H13	Module	-0.530543835	4.15005E-07
ZC3H13	Osteoporosis	-0.422151899	9.60356E-05
ZC3H13	Ossification	-0.081129864	0.474357274
ZC3H13	Bone_Development	-0.390365682	0.000343959
ZC3H13	Bone_Remodeling	-0.279301453	0.012108127
ZC3H13	Regulation_Of_Bone_Remodeling	0.018166901	0.872923985
ZC3H13	Osteoblast_Development	-0.339076418	0.002092663
ZC3H13	Regulation_Of_Osteoblast_Differentiation	-0.279535865	0.012033136
ZC3H13	Regulation_Of_Osteoblast_Proliferation	0.3	0.006858718
ZC3H13	Osteoclast_Differentiation	-0.0407173	0.719891644
ZC3H13	Multinuclear _Osteoclast_Differentiation	-0.367651195	0.000793827
ZC3H13	Osteoclast_Signaling	-0.395124238	0.000286447

ZC3H13	Regulation_Of_Osteoclast_Differentiation	0.007571496	0.946855072
FTO	Module	-0.272364465	0.01451897
FTO	Osteoporosis	-0.050762135	0.654750115
FTO	Ossification	-0.326935812	0.003076585
FTO	Bone_Development	-0.151032296	0.181125176
FTO	Bone_Remodeling	-0.21113579	0.060109782
FTO	Regulation_Of_Bone_Remodeling	-0.016291704	0.885947795
FTO	Osteoblast_Development	-0.186381777	0.097853122
FTO	Regulation_Of_Osteoblast_Differentiation	-0.200587205	0.074415603
FTO	Regulation_Of_Osteoblast_Proliferation	-0.041526263	0.714563291
FTO	Osteoclast_Differentiation	-0.122480793	0.279100969
FTO	Multinuclear _Osteoclast_Differentiation	-0.340930268	0.001970391
FTO	Osteoclast_Signaling	-0.021894174	0.84714028
FTO	Regulation_Of_Osteoclast_Differentiation	0.034048488	0.764304626
YTHDC1	Module	-0.499882794	2.34772E-06
YTHDC1	Osteoporosis	-0.27257384	0.014440542
YTHDC1	Ossification	-0.38448195	0.000429654
YTHDC1	Bone_Development	-0.349835912	0.001468004
YTHDC1	Bone_Remodeling	-0.353375527	0.001302836
YTHDC1	Regulation_Of_Bone_Remodeling	-0.10766526	0.341808356
YTHDC1	Osteoblast_Development	-0.476582278	7.86384E-06
YTHDC1	Regulation_Of_Osteoblast_Differentiation	-0.49273324	3.4344E-06

YTHDC1	Regulation_Of_Osteoblast_Proliferation	0.160993905	0.153685137
YTHDC1	Osteoclast_Differentiation	-0.209165495	0.06259704
YTHDC1	Multinuclear _Osteoclast_Differentiation	-0.393248945	0.000307965
YTHDC1	Osteoclast_Signaling	0.0407173	0.719891644
YTHDC1	Regulation_Of_Osteoclast_Differentiation	-0.092991092	0.411972661
YTHDC2	Module	-0.622292546	7.18454E-10
YTHDC2	Osteoporosis	-0.28851383	0.009448808
YTHDC2	Ossification	-0.442803563	3.90574E-05
YTHDC2	Bone_Development	-0.447046414	3.22297E-05
YTHDC2	Bone_Remodeling	-0.27238631	0.01451077
YTHDC2	Regulation_Of_Bone_Remodeling	-0.040014065	0.724534479
YTHDC2	Osteoblast_Development	-0.459845288	1.77715E-05
YTHDC2	Regulation_Of_Osteoblast_Differentiation	-0.538138772	2.62988E-07
YTHDC2	Regulation_Of_Osteoblast_Proliferation	0.360056259	0.001036154
YTHDC2	Osteoclast_Differentiation	-0.039943741	0.724999312
YTHDC2	Multinuclear _Osteoclast_Differentiation	-0.38488045	0.000423284
YTHDC2	Osteoclast_Signaling	-0.183684951	0.1028944
YTHDC2	Regulation_Of_Osteoclast_Differentiation	0.012330052	0.913558041
YTHDF1	Module	-0.260032818	0.019836549
YTHDF1	Osteoporosis	-0.009353024	0.934375433
YTHDF1	Ossification	-0.057571496	0.611978125
YTHDF1	Bone_Development	0.036779184	0.746017344

YTHDF1	Bone_Remodeling	-0.045335209	0.689661831
YTHDF1	Regulation_Of_Bone_Remodeling	0.059259259	0.60156711
YTHDF1	Osteoblast_Development	0.080028129	0.480399088
YTHDF1	Regulation_Of_Osteoblast_Differentiation	0.016572902	0.883992774
YTHDF1	Regulation_Of_Osteoblast_Proliferation	0.057852789	0.610237533
YTHDF1	Osteoclast_Differentiation	0.177191749	0.115862947
YTHDF1	Multinuclear _Osteoclast_Differentiation	-0.070065635	0.536850462
YTHDF1	Osteoclast_Signaling	-0.166830755	0.13911836
YTHDF1	Regulation_Of_Osteoclast_Differentiation	0.240248476	0.031827197
YTHDF2	Module	-0.513759962	1.09424E-06
YTHDF2	Osteoporosis	-0.365916549	0.000844111
YTHDF2	Ossification	-0.084692921	0.4551011
YTHDF2	Bone_Development	-0.453539616	2.38995E-05
YTHDF2	Bone_Remodeling	-0.14517112	0.198850747
YTHDF2	Regulation_Of_Bone_Remodeling	-0.009892171	0.930601677
YTHDF2	Osteoblast_Development	-0.263080169	0.018387477
YTHDF2	Regulation_Of_Osteoblast_Differentiation	-0.309610877	0.005195077
YTHDF2	Regulation_Of_Osteoblast_Proliferation	0.437365213	4.97802E-05
YTHDF2	Osteoclast_Differentiation	0.125269573	0.268216535
YTHDF2	Multinuclear _Osteoclast_Differentiation	-0.087529301	0.440086084
YTHDF2	Osteoclast_Signaling	-0.112869198	0.318845056
YTHDF2	Regulation_Of_Osteoclast_Differentiation	0.068659165	0.545075412

YTHDF3	Module	-0.57641819	2.2039E-08
YTHDF3	Osteoporosis	-0.500281294	2.29788E-06
YTHDF3	Ossification	-0.274941397	0.013578882
YTHDF3	Bone_Development	-0.610056259	1.8892E-09
YTHDF3	Bone_Remodeling	-0.308345054	0.005391461
YTHDF3	Regulation_Of_Bone_Remodeling	-0.058860759	0.604018194
YTHDF3	Osteoblast_Development	-0.646858884	9.03697E-11
YTHDF3	Regulation_Of_Osteoblast_Differentiation	-0.518963901	8.14647E-07
YTHDF3	Regulation_Of_Osteoblast_Proliferation	0.242662916	0.030096962
YTHDF3	Osteoclast_Differentiation	-0.188209095	0.094548707
YTHDF3	Multinuclear _Osteoclast_Differentiation	-0.583591186	1.33596E-08
YTHDF3	Osteoclast_Signaling	-0.147140178	0.192762322
YTHDF3	Regulation_Of_Osteoclast_Differentiation	-0.104805438	0.354858626
HNRNPC	Module	-0.169081106	0.133789677
HNRNPC	Osteoporosis	-0.166619784	0.139626024
HNRNPC	Ossification	-0.325152368	0.003251607
HNRNPC	Bone_Development	-0.257430849	0.021150158
HNRNPC	Bone_Remodeling	-0.23124707	0.039032135
HNRNPC	Regulation_Of_Bone_Remodeling	-0.023464604	0.836322953
HNRNPC	Osteoblast_Development	-0.312470699	0.004774554
HNRNPC	Regulation_Of_Osteoblast_Differentiation	-0.187458978	0.095894403
HNRNPC	Regulation_Of_Osteoblast_Proliferation	-0.295335209	0.00782358

HNRNPC	Osteoclast_Differentiation	-0.403094233	0.000209535
HNRNPC	Multinuclear _Osteoclast_Differentiation	-0.36411158	0.000899493
HNRNPC	Osteoclast_Signaling	0.08246601	0.46708524
HNRNPC	Regulation_Of_Osteoclast_Differentiation	-0.154453821	0.171324895

Supplementary Table 6. Information of m6A modification patterns.

ID	Cluster
GSM1369796	C1
GSM1369797	C1
GSM1369798	C1
GSM1369799	C2
GSM1369800	C1
GSM1369801	C2
GSM1369802	C1
GSM1369803	C1
GSM1369804	C1
GSM1369805	C2
GSM1369806	C2
GSM1369807	C1
GSM1369808	C1
GSM1369809	C1
GSM1369810	C2

GSM1369811	C1
GSM1369812	C1
GSM1369813	C1
GSM1369814	C1
GSM1369815	C2
GSM1369816	C1
GSM1369817	C1
GSM1369818	C1
GSM1369819	C2
GSM1369820	C1
GSM1369821	C2
GSM1369822	C2
GSM1369823	C2
GSM1369824	C2
GSM1369825	C1
GSM1369826	C2
GSM1369827	C2
GSM1369828	C1
GSM1369829	C2
GSM1369830	C2
GSM1369831	C2
GSM1369832	C2

GSM1369833	C1
GSM1369834	C1
GSM1369835	C2

Supplementary Table 7. Diversity of m6A regulators between cluster1 and cluster2.

Tag	FC	AveExpr	t	*P.* Value	adj.*P.* Val	B
YTHDF3	2.70442945	9.3320805	7.012388154	4.05E-09	5.34E-08	10.61471928
RBM15	2.644707682	7.8744055	6.842405773	7.63E-09	5.34E-08	9.99257574
WTAP	2.316944907	9.34361475	6.054364242	1.42E-07	6.64E-07	7.125897385
YTHDC2	2.027491256	6.894868	4.906718669	9.01E-06	3.15E-05	3.085875486
YTHDF2	1.502603143	9.11086325	2.899498193	0.005404665	0.015133062	-2.992020439
HNRNPC	1.371483081	11.13823975	2.625327924	0.011252873	0.024720868	-3.660084316
RBM15B	0.737022605	8.46971	-2.589019393	0.012360434	0.024720868	-3.744769205
YTHDC1	1.287553108	9.6707925	2.246529184	0.028806092	0.050410661	-4.497199239
METTL3	1.289944155	8.730059	1.889222716	0.064268442	0.099973132	-5.186900618
METTL16	1.149598964	5.5244055	1.592957752	0.117041552	0.163858173	-5.679144737
FTO	1.170239349	8.03428275	1.433479344	0.157519934	0.200479916	-5.912579441
ZC3H13	1.115960929	7.9111625	0.810636479	0.42115385	0.47695793	-6.600287458
CBLL1	0.913716175	5.559444	-0.773039789	0.442889506	0.47695793	-6.629977861
YTHDF1	1.062460834	9.129271	0.532055887	0.596882554	0.596882554	-6.787355847

Supplementary Table 8. Diversity of bone metabolism-related gene sets between cluster1 and cluster2.

Tag	logFC	AveExpr	t	*P*. Value	adj.*P*. Val	B
Multinuclear _Osteoclast _Differentiation	-0.085239195	0.0029189	-4.951167851	1.38E-05	0.000178806	1.987515726
Bone_Development	-0.012983915	0.028891657	-4.591564009	4.29E-05	0.000278705	0.868939081
Osteoblast _Development	-0.053823555	-0.500420286	-4.194668022	0.000146755	0.000635938	-0.334137467
Module	-0.025130756	-0.405260929	-4.027244358	0.000244184	0.000793597	-0.829227677
Regulation_Of _Osteoblast _Differentiation	-0.014274198	0.006907333	-3.894252522	0.000364108	0.000946681	-1.21639264
Ossification	-0.007473053	0.019970814	-3.355694331	0.001740987	0.003772138	-2.718823518
Osteoclast _Differentiation	-0.010432288	0.336972824	-2.415927191	0.020340628	0.037775451	-5.007809033
Osteoporosis	-0.005144936	0.096347107	-2.038453819	0.048135356	0.078219954	-5.772685147
Bone_Remodeling	-0.005592387	0.06492363	-1.341669971	0.187250809	0.270473391	-6.894511544
Regulation_Of _Osteoclast _Differentiation	-0.005791896	0.266442115	-1.045968897	0.301838828	0.392390477	-7.243134675
Regulation_Of _Osteoblast _Proliferation	0.005714518	0.016893925	0.719350875	0.476095821	0.562658698	-7.532310174
Osteoclast_Signaling	-0.002462553	-0.036375778	-0.400921622	0.690605897	0.748156388	-7.713286144
Regulation_Of_Bone _Remodeling	0.00035946	-0.110020194	0.054705416	0.956644771	0.956644771	-7.793826691

Supplementary Table 9. Univariate logistic regression results.

ID	OR	Low95	High95	*P* value
METTL3	0.149	0.022	1.01	0.051
METTL16	0.00026	1E-06	0.046749	0.022
WTAP	1.024	0.231	4.547	0.975
RBM15	1.39	0.322	5.99	0.659
RBM15B	7.315	0.524	102.204	0.139
CBLL1	48.649	2.805	843.734	0.008
ZC3H13	2.427	0.301	19.561	0.405
YTHDF1	2.223	0.148	33.402	0.563
YTHDF2	31.209	3.427	284.179	0.002
YTHDF3	1.787	0.483	6.61	0.384
YTHDC1	0.209	0.018	2.481	0.215
YTHDC2	1.006	0.214	4.724	0.994
HNRNPC	0.061	0.005	0.792	0.032
FTO	0.008	0.00023	0.279	0.008

Supplementary Table 10. Multivariate logistic regression results.

ID	OR	Low95	High95	*P* value
METTL16	0.002	0.000005	0.949	0.048
CBLL1	35.222	1.059	1170.98	0.046

YTHDF2	12.432	1.061	145.685	0.045
FTO	0.014	0.000211	0.95	0.047
HNRNPC	0.419	0.006	28.545	0.686

Supplementary Table 11. Risk scores of the samples.

ID	Risk Score
GSM1369756	-5.098792995
GSM1369757	-6.858377662
GSM1369758	-4.072686443
GSM1369759	-5.685455646
GSM1369760	-3.686368431
GSM1369761	-5.008415842
GSM1369762	-3.915904443
GSM1369763	-3.227826625
GSM1369764	-4.342389236
GSM1369765	-6.841712422
GSM1369766	-4.775632299
GSM1369767	-4.036042616
GSM1369768	-4.487024623
GSM1369769	-3.641029601
GSM1369770	-4.544843539
GSM1369771	-5.334891343

GSM1369772	-2.943638107
GSM1369773	-4.884059778
GSM1369774	-4.316736645
GSM1369775	-3.865590242
GSM1369776	-4.696163915
GSM1369777	-3.507975838
GSM1369778	-3.356147666
GSM1369779	-4.235975264
GSM1369780	-3.343992953
GSM1369781	-3.797793654
GSM1369782	-5.244537759
GSM1369783	-5.260549565
GSM1369784	-3.021930586
GSM1369785	-4.801883809
GSM1369786	-5.665344553
GSM1369787	-4.592955883
GSM1369788	-4.862480102
GSM1369789	-4.047520146
GSM1369790	-3.649585991
GSM1369791	-3.72880454
GSM1369792	-3.723141338
GSM1369793	-4.532126488

GSM1369794	-5.190432957
GSM1369795	-5.19991402
GSM1369796	-1.996199259
GSM1369797	-4.413510253
GSM1369798	-2.057692096
GSM1369799	-3.563086323
GSM1369800	-2.712222199
GSM1369801	-2.278908779
GSM1369802	-4.570734264
GSM1369803	-2.103540517
GSM1369804	-3.544544001
GSM1369805	-2.027569415
GSM1369806	-2.210443267
GSM1369807	-3.121396622
GSM1369808	-2.335903826
GSM1369809	-3.169890881
GSM1369810	-3.053204053
GSM1369811	-2.807664177
GSM1369812	-2.404998249
GSM1369813	-3.448494965
GSM1369814	-2.577161601
GSM1369815	-3.970587128

GSM1369816	-3.434208367
GSM1369817	-2.869874368
GSM1369818	-2.918272811
GSM1369819	-2.262470853
GSM1369820	-3.501876383
GSM1369821	-2.230211771
GSM1369822	-2.680715835
GSM1369823	-2.558738248
GSM1369824	-3.152101547
GSM1369825	-2.236075064
GSM1369826	-2.570394522
GSM1369827	-2.701027031
GSM1369828	-3.284979602
GSM1369829	-5.297508651
GSM1369830	-4.674779254
GSM1369831	-3.027632066
GSM1369832	-3.522925579
GSM1369833	-4.367061018
GSM1369834	-4.482970247
GSM1369835	-4.310812404

Supplementary Table 12. 306 target genes of the four m6A regulators coregulated from M6A2Target database.

Gene	Gene	Gene	Gene	Gene	Gene
OVGP1	TST	PLEKHG4B	DPYSL4	CORO1A	FAM105A
MAP2K1	SLC35G2	MAP7	ZNF687	RP1-179N16.6	PCDHGA1
ZNF213	DUSP4	TNNT1	ARHGAP5	FAM167A	GMDS-AS1
NES	BHLHE40	SERINC2	HMG20B	CH507-513H4.5	ZBED9
YJEFN3	HMOX1	HIST1H1C	SLC24A1	PPM1N	SRGAP3
AMH	JAGN1	PIK3CD	CBX7	DMRTA1	CBSL
ID1	RP11-804H8.6	KIAA0040	TXNRD3	RAB11B-AS1	LINC00205
NUPR1	KLRG1	SAMD9	KCNH2	FUT1	POLN
SYNM	SLC5A2	BACE2	IL17RC	MUC1	ZFYVE28
VMP1	NEU4	MAPK13	JOSD2	KCTD12	FAT4
TGM2	IFITM10	ZNF385A	TTYH3	HES7	PRR5
SNAI1	MAFB	HIST1H4I	CDC42BPG	HLA-F-AS1	MTHFR
KLHL17	SLC43A2	SH3BP1	SMARCC2	ZNF467	IGSF3
ADRM1	RGS3	TOP1MT	KIF3C	SGMS1-AS1	RP11-403I13.8
MEF2D	PTP4A3	ABHD14B	PALD1	USP46-AS1	NAV1
LIMD2	GEM	LAMA5	EGFLAM	DLG2	ZNF678
KCNJ14	YPEL5	CLSTN3	FJX1	TSHZ2	PSD4
ST3GAL3	OLFM1	BNIP3	MYD88	SP6	CPNE1
KLHL15	ADRA1B	SPATA20	SGK223	RP11-115C21.2	STX16
SPHK1	BCL2	H1F0	WNT7B	KLLN	HELZ2
ID3	Metazoa_SRP	KIF21B	TRIM65	IL4I1	ZBTB46

SH2B2	DDR2	SON	PRRT3	CXXC4	TCEA2
SH3KBP1	NDUFA13	CHMP3	ALS2CL	CHSY3	SLC6A8
CD27-AS1	LINC00346	EPB41L1	LIPT2	HOXA6	ANKRD16
SHISA4	CPA4	PLCG1	EDA	TBXAS1	STK32C
CSF1	MAFG-AS1	ASAP3	UBE2L6	RP11-798M19.6	MACROD1
SCNN1D	PLIN2	DLX4	AATK	DBP	ANKRD13D
LINC00634	SLC25A25-AS1	SRCIN1	CD24	USP32P3	NDUFS8
GPR153	GDF15	MBOAT7	STS	TLE6	RAPGEF3
SPSB1	KIAA1683	RTN4R	SLC9A3	URGCP-MRPS24	PA2G4
NEIL2	TNFSF9	ARL10	GCH1	ZNF117	ZADH2
SLC6A9	SYT17	NGEF	TMEM231	CTBP1	POLR2E
EMP1	C10orf54	TNS3	TRANK1	PIGV	STK11
SOCS3	CYP27A1	NOXA1	GLB1L	ZNFX1	CD320
ATP1B3	GPSM3	C1orf198	DHRS4-AS1	NR1H3	MARK4
PROSER2	SLC16A6	FAM110C	DYRK3	FUT8	SIX5
SERTAD1	AEBP1	TUBA1A	CMTR2	TMEM8A	TM9SF1
TNFRSF10D	KIF26B	P3H4	NKX2-8	LMF1	VASH1
ECM1	SERPINF1	PRMT6	PARP12	LA16c-306E5.2	NRDE2
SPRY4	IER3	CD82	FENDRR	CCDC189	MAPK8IP3
HTRA3	PLEKHA6	TMEM121	JPH1	LRRC75A	CASKIN1
FBN1	WNT6	MANSC1	CSPG5	TTC33	PHKG2
NKD1	ALPK2	TCF19	HPSE	LLfos-48D6.2	C16orf74

WNT5B	AZU1	POLG	DTD2	CDR2	ZNF469
PLCB4	JUNB	PIH1D1	DDX60	KANK1	SLC5A10
APLN	KLHDC7B	RARG	FOXL2	FAM129B	SLC25A39
BCAR3	RP11-231C14.7	LRFN3	IFIT1	WIPI2	KCNJ2
SCNM1	DERL3	LPL	STAB1	NUDCD3	GPRC5C
FAM86B3P	APLP1	RAD23A	MCF2L2	CLDN15	MAD1L1
ZBTB43	COL1A1	TM7SF2	ISG15	C7orf49	AHRR
RP11-552M11.8	EGFL7	GSS	OBSL1	DOCK5	OCEL1

Supplementary Table 13. The description of 26 targets of m6A regulators from the Ensembl database.

Gene	Ensembl	Gene	Ensembl
TGM2	ENSG00000198959	JUNB	ENSG00000171223
SNAI1	ENSG00000124216	COL1A1	ENSG00000108821
MEF2D	ENSG00000116604	ZNF385A	ENSG00000161642
ID3	ENSG00000117318	P3H4	--
CSF1	ENSG00000184371	POLG	ENSG00000140521
ECM1	ENSG00000143369	RARG	ENSG00000172819
SPRY4	ENSG00000187678	WNT7B	ENSG00000188064
FBN1	ENSG00000166147	HPSE	ENSG00000173083
MAFB	ENSG00000204103	ISG15	ENSG00000187608
BCL2	ENSG00000171791	CTBP1	ENSG00000159692
DDR2	ENSG00000162733	FAT4	ENSG00000196159

CYP27A1	ENSG00000135929	PHKG2	ENSG00000156873
AEBP1	ENSG00000106624	ZNF469	ENSG00000225614

Supplementary Table 14. Information of GO/KEGG analysis.

Ontology	Description	Gene Ratio	Bg Ratio	*P*. Value	adj.*P*. Val	q value	Gene ID	Count
BP	ossification	12/26	398/18670	5.51277E-14	6.62E-11	4.25354E-11	BCL2/COL1A1/CSF1/ECM1/ID3/JUNB/MEF2D/DDR2/SNAI1/WNT7B/ISG15/FAT4	12
BP	regulation of ossification	7/26	203/18670	8.94382E-09	1.07236E-05	3.45043E-06	BCL2/CSF1/ECM1/ID3/DDR2/WNT7B/ISG15	7
BP	osteoblast differentiation	7/26	225/18670	1.8207E-08	2.1812E-05	4.68272E-06	COL1A1/ID3/JUNB/MEF2D/DDR2/SNAI1/WNT7B	7
BP	connective tissue development	7/26	273/18670	6.86478E-08	8.21714E-05	1.32418E-05	COL1A1/CSF1/ECM1/MEF2D/RARG/SNAI1/WNT7B	7
BP	metanephros development	5/26	90/18670	1.41281E-07	0.000168972	2.18019E-05	BCL2/FBN1/ID3/WNT7B/FAT4	5
BP	cartilage development	6/26	209/18670	3.49814E-07	0.000418028	4.49849E-05	COL1A1/ECM1/MEF2D/RARG/SNAI1/WNT7B	6
BP	bone development	6/26	217/18670	4.36191E-07	0.000520812	4.80794E-05	COL1A1/FBN1/MEF2D/RARG/ZNF385A/FAT4	6
BP	collagen fibril organization	4/26	54/18670	8.91168E-07	0.001063164	8.59509E-05	AEBP1/COL1A1/DDR2/P3H4	4
BP	myeloid cell differentiation	7/26	416/18670	1.18253E-06	0.001409573	0.000101379	CSF1/FBN1/JUNB/RARG/ISG15/MAFB/ZNF385A	7

BP	embryonic organ development	7/26	428/18670	1.42948E-06	0.001702507	0.000110295	FBN1/ID3/JUNB/RARG/SNAI1/WNT7B/MAFB	7
BP	urogenital system development	6/26	330/18670	4.97742E-06	0.005923126	0.000349134	BCL2/FBN1/ID3/RARG/WNT7B/FAT4	6
BP	osteoclast differentiation	4/26	97/18670	9.37537E-06	0.011147319	0.000578767	CSF1/FBN1/JUNB/MAFB	4
BP	osteoblast proliferation	3/26	31/18670	1.05015E-05	0.012475791	0.000578767	BCL2/JUNB/HPSE	3
BP	negative regulation of intrinsic apoptotic signaling pathway in response to DNA damage	3/26	31/18670	1.05015E-05	0.012475791	0.000578767	BCL2/SNAI1/ZNF385A	3
BP	negative regulation of signal transduction by p53 class mediator	3/26	33/18670	1.27231E-05	0.015089624	0.00065446	BCL2/SNAI1/ZNF385A	3
BP	regulation of intrinsic apoptotic signaling pathway in response to DNA damage	3/26	39/18670	2.11939E-05	0.025114748	0.000944566	BCL2/SNAI1/ZNF385A	3
BP	gland morphogenesis	4/26	120/18670	2.17502E-05	0.025752216	0.000944566	BCL2/CSF1/RARG/TGM2	4
BP	regulation of myeloid cell differentiation	5/26	251/18670	2.20356E-05	0.026068082	0.000944566	CSF1/FBN1/RARG/ISG15/MAFB	5
BP	gland development	6/26	434/18670	2.36434E-05	0.027946551	0.000960147	BCL2/CSF1/RARG/TGM2/WNT7B/MAFB	6

BP	endochondral bone growth	3/26	44/18670	3.05721E-05	0.036105616	0.001179438	ECM1/DDR2/RARG	3
BP	kidney development	5/26	278/18670	3.59442E-05	0.042414138	0.001309112	BCL2/FBN1/ID3/WNT7B/FAT4	5
BP	bone growth	3/26	47/18670	3.73267E-05	0.044008127	0.001309112	ECM1/DDR2/RARG	3
BP	renal system development	5/26	293/18670	4.61753E-05	0.05439447	0.001549038	BCL2/FBN1/ID3/WNT7B/FAT4	5
BP	biomineral tissue development	4/26	163/18670	7.2059E-05	0.084813431	0.002316633	COL1A1/ECM1/DDR2/ISG15	4
BP	collagen-activated tyrosine kinase receptor signaling pathway	2/26	10/18670	8.33455E-05	0.098014298	0.002572305	COL1A1/DDR2	2
CC	collagen-containing extracellular matrix	5/26	406/19717	0.000166262	0.013300973	0.010325755	AEBP1/COL1A1/ECM1/FBN1/TGM2	5
CC	endoplasmic reticulum lumen	4/26	309/19717	0.00067359	0.053213607	0.020916741	COL1A1/CSF1/FBN1/WNT7B	4
CC	transcription factor complex	4/26	365/19717	0.001251097	0.097585543	0.021883769	CTBP1/JUNB/RARG/MAFB	4
MF	protease binding	4/25	128/17697	2.93598E-05	0.002935978	0.002286972	BCL2/COL1A1/ECM1/POLG	4
MF	extracellular matrix structural constituent	4/25	163/17697	7.5449E-05	0.007469455	0.002938541	AEBP1/COL1A1/ECM1/FBN1	4
MF	transcription corepressor activity	3/26	238/17697	0.000323095	0.031663309	0.008389133	AEBP1/CTBP1/ID3/JUNB	4

KEGG	Parathyroid hormone synthesis, secretion, and action	3/26	106/7914	0.001385384	0.110830701	0.097706013	MEF2D/MAFB/BCL2	3
KEGG	Proteoglycans in cancer	3/26	204/7914	0.008788598	0.267192975	0.235551701	COL1A1/WNT7B/HPSE	3
KEGG	Human papillomavirus infection	3/26	330/7914	0.031640627	0.267192975	0.235551701	COL1A1/WNT7B/ISG15	3
KEGG	PI3K-Akt signaling pathway	3/26	354/7914	0.03785497	0.267192975	0.235551701	CSF1/BCL2 /COL1A1	3
KEGG	Pathways in cancer	3/26	530/7914	0.100842167	0.359881498	0.317263952	BCL2/WNT7B/CTBP1	3
KEGG	TGF-beta signaling pathway	2/26	94/7914	0.016905718	0.267192975	0.235551701	ID3/FBN1	2
KEGG	AGE-RAGE signaling pathway in diabetic complications	2/26	100/7914	0.019001314	0.267192975	0.235551701	BCL2/COL1A1	2
KEGG	TNF signaling pathway	2/26	112/7914	0.023504174	0.267192975	0.235551701	CSF1/JUNB	2
KEGG	Osteoclast differentiation	2/26	128/7914	0.030123182	0.267192975	0.235551701	CSF1/JUNB	2
KEGG	Signaling pathways regulating pluripotency of stem cells	2/26	140/7914	0.035522156	0.267192975	0.235551701	ID3/WNT7B	2
KEGG	Gastric cancer	2/26	149/7914	0.039802506	0.267192975	0.235551701	BCL2/WNT7B	2
KEGG	Wnt signaling pathway	2/26	160/7914	0.04529003	0.278707877	0.245702997	WNT7B/CTBP1	2

2. Supplementary Figures

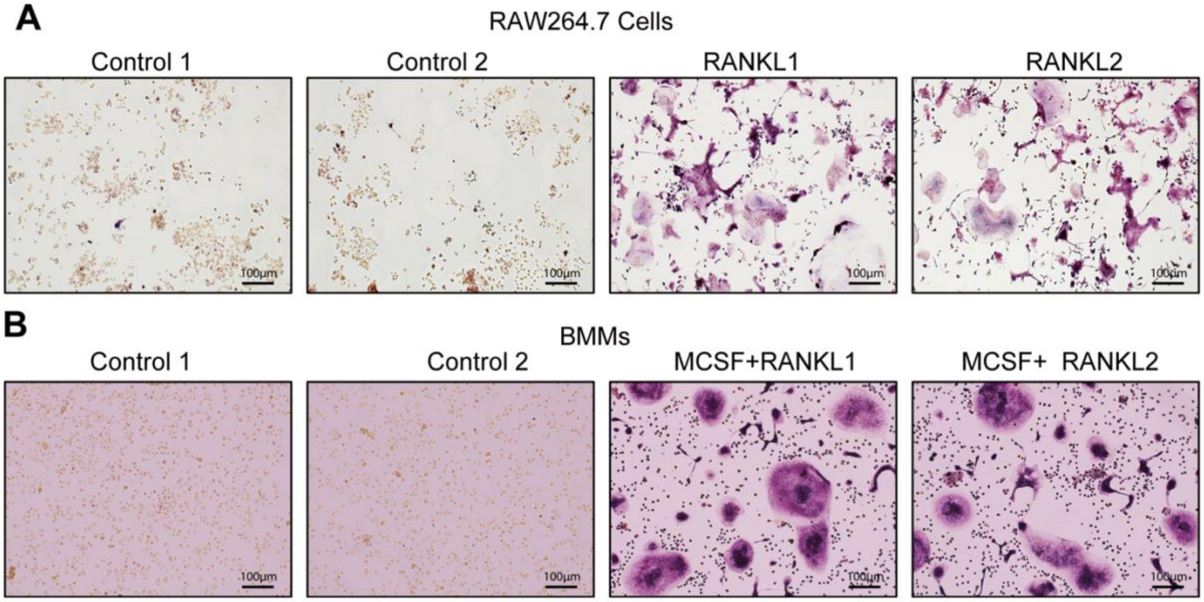

Supplementary Figure1. The repeated images of TRAP staining of RAW 264.7 cells (A) and BMMs(B) before and after osteoclasts differentiation.
